# Water quality trend assessment in Jakarta: A rapidly growing Asian megacity

**DOI:** 10.1371/journal.pone.0219009

**Published:** 2019-07-11

**Authors:** Pingping Luo, Shuxin Kang, Apip, Meimei Zhou, Jiqiang Lyu, Siti Aisyah, Mishra Binaya, Ram Krishna Regmi, Daniel Nover

**Affiliations:** 1 Key Laboratory of Subsurface Hydrology and Ecological Effects in Arid Region, Ministry of Education, Chang’an University, Xi’an, China; 2 School of Environmental Science and Engineering, Chang’an University, Xi'an, China; 3 Research Centre for Limnology, Indonesian Institute of Sciences (LIPI), Jakarta, Indonesia; 4 Faculty of Science and Technology, Pokhara University, Pokhara, Nepal; 5 Environment & Resource Management Consultant (P) Ltd., New Baneshwor, Kathmandu, Nepal; 6 School of Engineering, University of California–Merced, Merced, CA, United States of America; University of the Chinese Academy of Sciences, CHINA

## Abstract

Megacities are facing serious water pollution problems due to urbanization, rapid population growth and economic development. Water is an essential resource for human activities and socio-economic development and water quality in urban settings has important implications for human and environmental health. Urbanization and lack of sewerage has left the water in Jakarta, Indonesia in a heavily polluted condition. Rigorous assessment of urban water quality is necessary to understand the factors controlling water quality conditions. We use trend analysis to assess the current water quality conditions in Jakarta, focusing on Biochemical Oxygen Demand (BOD), Dissolved Oxygen (DO), and Total Suspended Solids (TSS). In most monitoring stations analyzed, BOD and TSS concentrations have decreased over time, but from large starting concentrations. DO in most monitoring stations has increased. Although Jakarta’s water quality has shown some improvement, it remains heavily impaired. The average value of BOD is low in upper stream stations compared to middle and lower stream stations. BOD and TSS trends of some water quality stations in middle and lower streams show increasing trends. Cluster analysis results suggest three groups for BOD and TSS, and four groups for DO. Understanding water quality conditions and factors that control water quality suggest strategies for improving water quality given current trends in climate, population growth and urban development. Results from this study suggest research directions and management strategies to address water quality challenges.

## 1. Introduction

Water pollution continues to be a major challenge in the context of urban development and population growth, particularly in contexts without adequate wastewater treatment [1–3]. Jakarta, Indonesia is a rapidly growing megacity facing water quality impairment due to pollution and a lack of wastewater treatment. The analysis presented here identifies causes of water quality degradation to support design of robust monitoring systems and sustainable water quality management strategies. These efforts will assist ongoing efforts by the government of Jakarta to improve water quality.

Trend analysis has been used in many applications, including air quality [4–6], hydrology [7–11], streamflow [12–16], rainfall [17–25]. Water quality trend analysis has been carried out in previous studies outside the context presented here. For example, water quality trend analysis was done for the Nakdong River of Korea [26], the Eymir Lake of Ankara, Turkey [27], the Atlantic Region of Canada [28], 92 major rivers of Japan [29], the Ebro River of Spain [30], the Gomti River of India [31], the Poyang Lake basin of China [32], the Strymon River in Greece [33], and the Kizilimak River of Turkey [34]. Past studies used trend analysis to understand water quality in river systems but not in urban settings. Trend analysis of water quality in urban areas can provide detailed information for resource managers to identify causes of and solutions for water pollution problems.

Environmental problems and sustainability have been studied in Jakarta to find strategies ensuring balanced economic, social, and environmental development [35]. Abidin et al. [36] studied land subsidence and its relationship with urban development, while Irawan et al. [37] researched water quality and the interaction between surface and groundwater in the Ciliwung River, Jakarta, Indonesia. Putri et al.[38] presented results focused on the impact of land use on groundwater quality in the Ciracas subdistrict, East Jakarta and Fulazzaky [39] used a water quality index to assess the water quality status of the Citarum River, Jakarta. Identifying integrated solutions for flooding and water quality problems in Jakarta is also a priority research area [40]. A national-level water quality analysis was conducted for Indonesia [41] but no detailed water quality research has been conducted for Jakarta.

This study uses spatiotemporal trend analysis of water quality to generate an overview of water quality for Jakarta. The overall objective of this research is to provide resources to improve water quality through designation of water usage, establishment of criteria to protect water resources, and development of water quality management plans. The results of this study provide an analytical foundation for stakeholders to better understand water quality conditions in urban megacities. Understanding water conditions in Jakarta will yield strategies for future urban water management in Jakarta and beyond.

## 2. Study sites, data and methodology

### 2.1 Study area and data

Jakarta is the capital and largest city of Indonesia, located on the northwest coast of Java with an area of 662 km^2^. Jakarta is administratively equal to a province (officially called the Special Capital City District of Jakarta—DKI Jakarta). About 9 million people live in the city, which is divided into five municipalities. The metropolitan area around Jakarta is called Jabodetabek and includes Jakarta, Bogor, Depok, Tanggerang and Bekasi, and based on 2015 rankings, is the second largest megacity in the world. Jakarta includes five districts which are central, west, east, south and north.

The elevation of Jakarta is from −2 to 50 meters with average elevation of 8 meters above sea level. About 40% of Jakarta’s area, notably in the north, is below sea level. Thirteen rivers pass through Jakarta, the largest of which is the Ciliwung. Jakarta is prone to flooding due to wet season rains and insufficient drainage. The wet season of Jabodetabek is from October to May with maximum rainfall generally observed in January and February during the peak of the monsoon. High intensity, short duration storms usually occur in this area. The afternoons and evenings experience 60%-80% of the rainfall. Floods occur annually in Jakarta, notably in 1996, 2002 and 2007 with up to 40% of the city inundated. Population growth and land subsidence in Jakarta are major factors contributing to increasing flood risk. Surface water pollution in Jakarta comes from residential and commercial wastewater, industry, agriculture, solid waste and leakage from septic tanks.

We present a research process for urban water quality assessment in Jakarta. Initially, previous research and government reports are collected. The research process begins with collection of urban growth data (e.g. population and land use) and observed water quality data. Second, statistical analysis is carried out to identify trends in urban water quality and urban water conditions. The review assessment is conducted to understand the urban water environment and identify the factors contributing to water pollution problems. Finally, statistical analysis and review assessments are used to make recommendations about sustainable water and environmental management.

**Fig 1:**
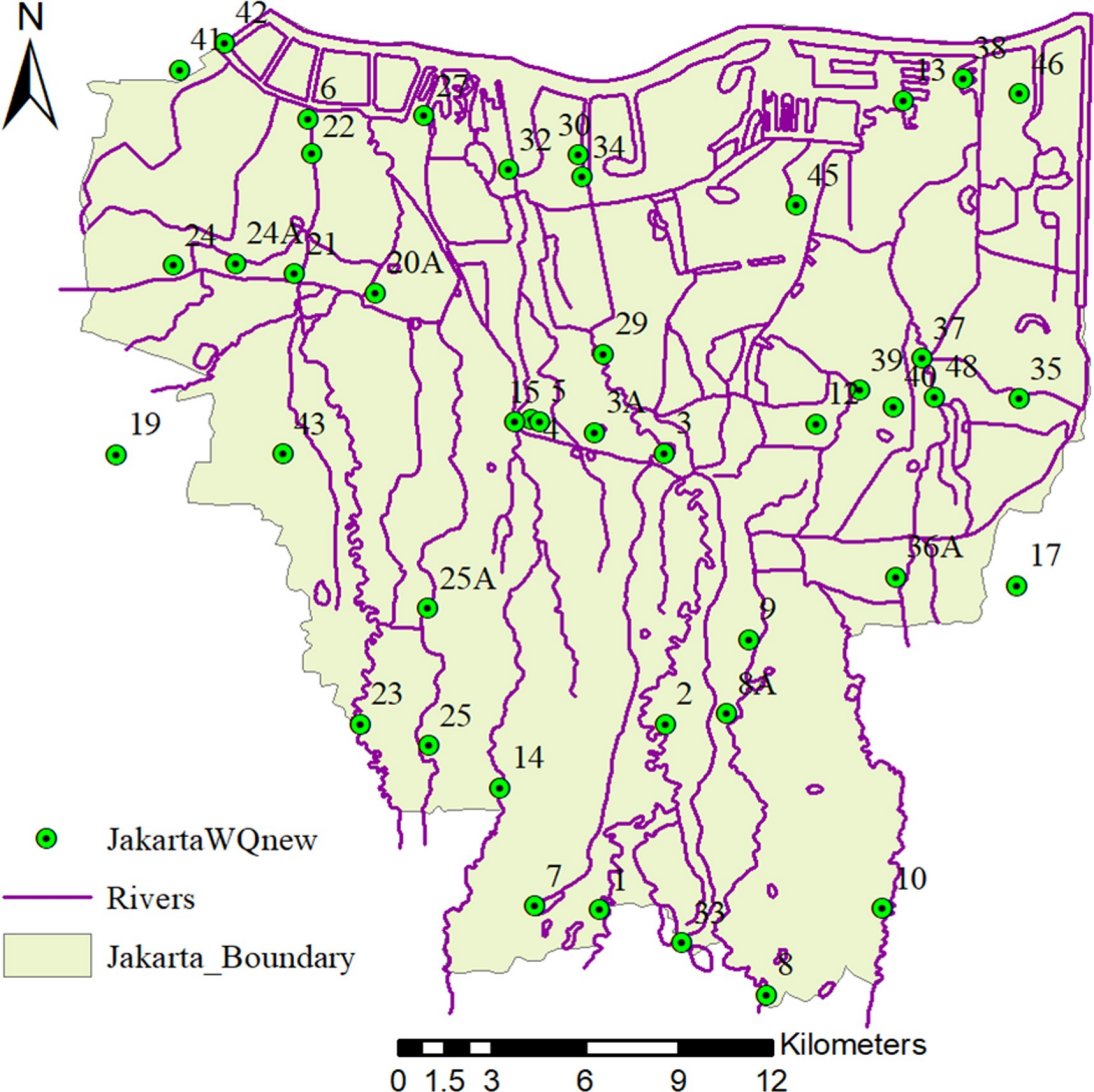
Location of river water quality monitoring stations conducted by the Government of DKI Jakarta Province (The river network and boundary map are drawn by Dr. Apip; The location map is drawn by Dr. Pingping Luo).

Secondary water quality data (26 instances from 2008 to 2014) were collected from the government of DKI Jakarta Province, the Ministry of Public Works, the Local Government of Jakarta Province and the Ministry of Environment and Forestry. Socio-economic data were collected from Statistics Indonesia and the Indonesian Institute of Sciences (LIPI). We investigate three water quality parameters: Biochemical Oxygen Demand (BOD), Dissolved Oxygen (DO), and Total Suspended Solid (TSS). We use 44 observed water quality stations (Fig 1) with data from 2008 to 2014 (Table 1).

**Table 1:** List of water sampling stations for secondary water quality data.

Station NO.	Station Name and location	Station NO.	Station Name and location
1	S. Ciliwung, Kelapa Dua (Srengseng Sawah)	24	Kali Mookervart, Jl. Daan Mogot, Bir Bintang (Kali Deres)
2	S. Ciliwung, Intake PAM Condet (kampungGedong)	24A	Kali Mookervart, Jl. Daan Mogot Pemancar (Rawa Buaya)
3	S. Ciliwung, Sblm Pintu Air Manggarai	25	S. Grogol, Jl. Lebak Bulus
3A	S. Ciliwung, Jl. Halimun	25A	S. Grogol, Jl. Radio Dalam
4	S. Ciliwung, Jl. KH. Mas Mansyur (Karet Tengsin)	27	S. Grogol, PLTU Pluit
5	S. Ciliwung, Gudang PLN (Kebon Melati)	29	S.Ciliwung, Jl. Kwitang
6	S. Ciliwung, Jemb Pantai Indah Kapuk	30	S. Ciliwung Gajah Mada, Ancol Marina
7	S. Kalibaru, Komp. Srengseng Sawah	32	S. Ciliwung Gajah Mada, Pompa Pluit
8	S. Cipinang, Jl. Auri (Taman Bunga Cibubur)	33	Sungai Kali Baru Timur, Jl. Raya Bogor (YKK)
8A	S. Cipinang, Jl. Pondok Gede Tol TMII (Kramatjati)	34	S. Kaali Baru Timur, Ancol/Jembatan si manis
9	S. Cipinang, Jl. Halim P Kusuma	35	S.Cakung, Jl. Pulogebang
10	Kali Sunter (Pondok Rangon)	36A	S. Buaran, Jl. Kalimalang (Pondok Kelapa)
12	S. Sunter, Jl. Jatinegara Kaum	37	Cakung Drain, Jl. Raya Bekasi (Cakung Barat)
13	Kali Sunter, Bogasari (Koja Selatan)	38	Cakung Drain, Cilincing (Pos Polisi)
14	S. Krukut, Pondok Labu	39	S. Petukangan, Kawasan PT. JIEP
15	S. Krukut, Jl. Pejompongan (Karet Tengsin)	40	S. Petukangan, Jl. Swadaya
17	Tarum Barat, Bekasi	41	S. Kama, Jl. Raya Benda
19	S. Angke, Cileduk	42	S. Kamal, Muara Kamal
20A	S. Angke, Pesing Kali Angke	43	S. Sepak,r Jl.Pasar Bintaro. Ulujami
21	Cengkareng Drain,Rel Kereta Api (Kembangan)	45	Kali Sunter, Komp. AL, Jl. Yos Sudarso (Kelapa Gading)
22	Cengkareng Drain, Kapuk (Muara Cengkareng Drain)	46	Kali Blencong, Muara Baru (Rorotan)
23	S. Pesanggerahan(Jl. Ciputat, Ps. Jumat, Lebak Bulus	48	S. Buaran, Belakang PIK

### 2.2 Methodology

In this study, the Mann-Kendall test (MKT) was applied to identify trends in urban water quality parameters. MKT is a non-parametric statistical procedure used to test for trends in time series data. For independent and randomly ordered data in a time-series *X*_*i*_ {*X*_*i*_, *i* = 1, 2, …, n}, the null hypothesis *H*_*0*_ is tested on the observations *X*_*i*_ against the alternative hypothesis *H*_*1*_, where there is an increasing or decreasing monotonic trend [42]. Gibert [43] divided *n* (number of values) <10 and *n*≥10 to calculate the normal approximation *Z*. In this study, the time series includes 26 events (n>10). According to the condition n ≥ 10, the S variance is described according to Eq (1) below:

$$\mathrm{Var}(S)=\frac{n(n‐1)(2n+5)‐\sum_{i=1}^{e}t_{f}(t_{i}‐1)(2t_{i}+5)}{18}$$ (1)

where, *e* is the number of whole groups and *t*_*i*_ is the number of data values in the *i*th group.

The statistical *S* test is given as follows:

$$S=\sum_{e=1}^{n‐1}\sum_{i=e+1}^{n}\mathrm{sgn}(x_{i}‐x_{e})$$ (2)

where,

$$\mathrm{sgn}(\varphi )=\left\{ \begin{array}{ll} 1 & \varphi >0\\ 0 & \varphi =0\\ ‐1 & \varphi <0 \end{array}\right.$$ (3)

The normal approximation Z test using the statistical value S and the variance value Var(S) is written in the following form:

$$Z=\left\{ \begin{array}{ccc} \frac{S‐1}{\sqrt{\mathrm{Var}(S)}} & \mathrm{if} & S>0\\ 0 & \mathrm{if} & S=0\\ \frac{S+1}{\sqrt{\mathrm{Var}(S)}} & \mathrm{if} & S<0 \end{array}\right.$$ (4)

For the normal approximation Z test, the Z value shows the statistical trend. If Z < 0, it indicates a decreasing trend and if Z > 0, it indicates an increasing trend.

Cluster analysis is the process of classifying data into different classes or clusters. This analysis suggests that objects in the same cluster have great similarity, possibly indicating similar pollution sources. Clustering analysis methods include system clustering, K-means clustering, and two-step clustering. This study adopts the method of systematic clustering. Calculating clustering distance index can be done using Euclidean distance, squared Euclidean distance, Block, Chebyshev distance, and other methods. Squared Euclidean distance was used in this study.

## 3. Overview of urban and water environment of Jakarta

### 3.1 Land use and urban development of Jakarta

Urban areas of Jakarta have limited green areas due to rapid development of tall structures, commercial buildings, industrial and residential areas during the mid-1990s [44]. WALHI, an Indonesian environmental NGO, reported that at least five designated green areas were converted to commercial areas and shopping malls in the Jakarta spatial plan of 1985–2005. The green area of Jakarta in 2013 is only about 10,008 hectares (9%) compared with the original amount in 2007 (33,467 hectares, 29%).

There are important feedbacks between environmental and social conditions. Rapid population growth and urban development continue to threaten the environment of Jakarta as people are attracted to economic opportunities in the city. Urbanization leads to increased residential areas and reduction in green spaces, which happened quickly from 2007 to 2013 [45]. Flood prevention requires ample green space as does safeguarding ecological and human health. Green spaces also contribute to improving groundwater quality, flood prevention, improving air quality and decreasing urban temperatures. Water demand in Jakarta is gradually increasing due to population growth and urban development, a trend that has not been addressed by increasing surface water supplies. Those without surface water access must use groundwater, extraction of which is driving land subsidence [46] and consequent increase in flood risk. Continuous land subsidence will endanger drainage structures in Jakarta and further exacerbate floods.

### 3.2 Water demand and water stress in Jakarta

**Fig 2:**
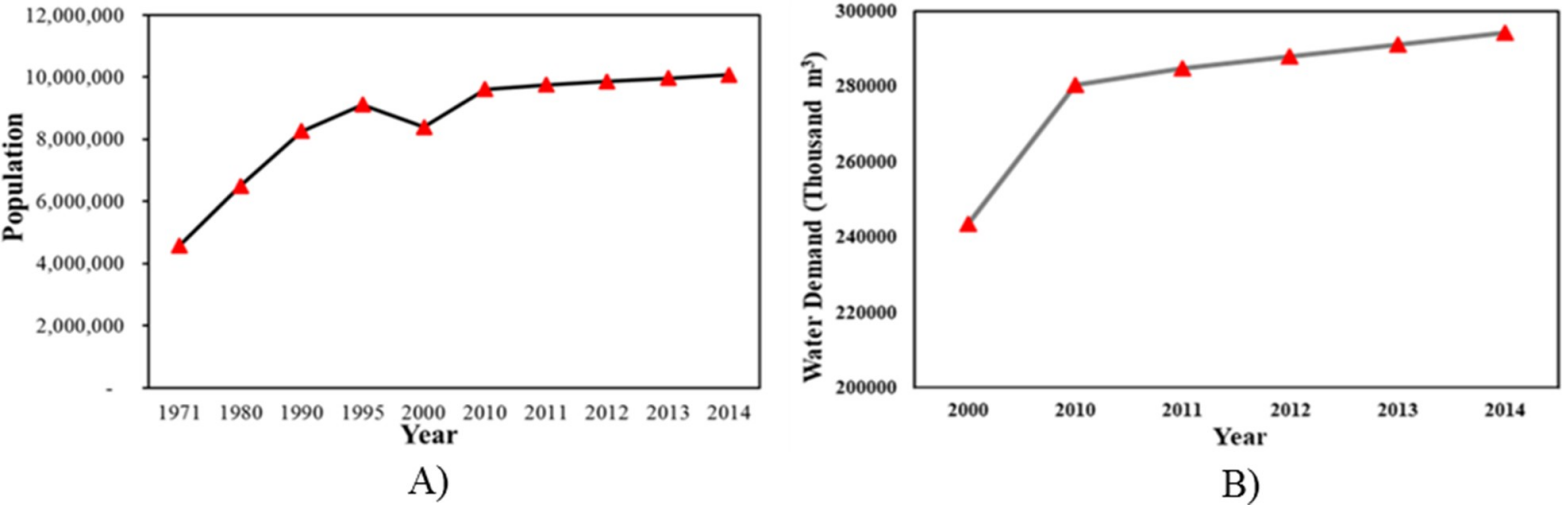
A) Population from 1971 to 2014 in Jakarta; B) Total demand for domestic water use in DKI Jakarta per year (thousand m^3^).

Jakarta’s population increased significantly from 1971 to 1995 (Fig 2A). From 1996 to 2000, population decreased and has been rising since 2000. As a consequence of population growth, water stress is becoming increasingly serious. Water demands in Jakarta are divided into domestic and non-domestic use. Domestic demand in Jakarta has increased from about 243 to 294 million m^3^ over the past fourteen years [47]. Fig 2B shows that the water demand in 2010 increased by 40 million m^3^ when compared to 2000, and water demand for domestic use maintained a significant increasing trend from 2010 to 2014. Jakarta’s water mainly comes from the Citarum River, the Jatiluhur Resrvoir and other areas outside of Jakarta. Groundwater is another, less dependable, water source for domestic needs. However, groundwater is limited in Jakarta and domestic uses must compete with the private sector.

Water shortages have plagued poor communities of Jakarta for years. Wealthy residential areas have piped water, but many poor communities and slums do not. Private water supply was planned to improve the water supply system in Jakarta, although socio-economic variability has presented a challenge. Lack of adequate funding for piped water supply means that poor communities are not able to afford clean water while middle-class communities receive clean water based on the description from the private company of PD PAL JAYA [48,49]. Poorer communities rely on polluted river water, lakes and shallow wells to meet their water needs while those who can afford to, buy water from kiosks and shops. Addressing the gap between supply and demand is required to achieve sustainable development of Jakarta.

### 3.3 Urban water environment

Urban rivers in Asian megacities are facing serious pollution problems from domestic, agricultural and industrial sources, partly due to lack of wastewater treatment exacerbated by population growth and development. Jakarta is no exception to these trends, as increasing population density and urban development increase water and wastewater treatment demand without concurrent increases in supply. Urban sewerage covers just 1.9% of Jakarta’s population [50]. Most of Jakarta’s population depends on on-site sanitation, but infrastructure is weak and drains directly to surface waters. The same is true for industrial wastewater in the city.

Jakarta lacks a comprehensive wastewater treatment plant. Wastewater treatment systems are divided into off-site management (sewerage) and on-site sanitation [51]. On-site sanitation was mainly applied in dispersed residential and slum areas. There are about 8.4 million people in Jakarta (71%) who collected their wastewater using individual septic tanks. About 1.3 million people (11%) in slum areas discharge their wastewater directly to the river without treatment. Off-site sanitation is mainly applied in apartments, office buildings, and industrial settings. About 1.9 million people (16%) depend on individual treatment plants (ITP), some with advanced treatment processes (ATP) before discharging to rivers. About 200,000 people use off-site sanitation, delivering wastewater through the sewerage system to the pumping station and Setiabudi Wastewater Treatment Plant (WWTP).

Sustainable wastewater management must entail systematic collaboration between government, industry, and other stakeholders. Wastewater management in Jakarta is managed by national and regional government and industry. The national department for wastewater management is guided by the National Planning Board in the Ministry of National Development Planning (BAPPENAS) and collaborates with the sector of Sewerage Planning & Construction at the Directorate General for Human Settlements (DGHS) of the Ministry of Public Works. The regional wastewater management is directed by the Planning Board of the Regional Planning and Development Agency (BAPPEDA) working with the Storm Water Drainage Management sector of DPU (Public Works Agency), the Environmental & Wastewater Management sector of BPLHD (Environmental Agency), and the Sludge Collecting & Treatment Service sector of DK (Cleansing Agency). Private wastewater management is led by PD PAL JAYA which provides the sewerage operation and maintenance service in Jakarta (see Table 2).

**Table 2:** English name and Indonesia full name of Indonesia acronym list.

Indonesia Acronym	Indonesia full name	English mean or name
DPU	Dinas Pekerjaan Umum	Public Works Agency
BAPPENAS	Badan Perencanaan Pembangunan Nasional	National Planning Board in the Ministry of National Development Planning
BAPPEDA	Badan Perencanaan Pembangunan Daerah	Regional Planning & Development Agency
DK	Dinas Kebersihan	Cleansing Agency
BPLHD	Badan Perlindingan Lingkungan Hidup Daerah	Regional Environmental Agency
PD PAL JAYA	Perusahaan Daerah	Regional Company

### 3.4 Managements for urban water

Rapid urban development in Jakarta increasingly requires management to solve water pollution problems. President Suharto led Indonesia from 1966 to 1998 and oversaw a period of rapid economic growth and political stability. Water pollution problems and high water demand have forced the Government of Jakarta to contrive an urban development plan with consideration of environmental health. Environment pollution control (waste, water, and air pollution) and conservation of protected areas are increasingly included in development planning for Jakarta. Mapping initiatives are increasingly assisting project execution to meet the needs for economic development, particularly to include environmental protection and ecosystem services [52].

Based on the ongoing Mid-Term Development Plan (2012–2017) of Jakarta, the government has set three major targets which relate to Jakarta’s environmental rehabilitation [53]:

1. Jakarta shall become a managed modern city with robust spatial planning, including several improvements in flood control systems, water supply systems, utilization of groundwater and wastewater treatment.
2. Jakarta shall be free of chronic problems such as traffic, floods, slums, and waste.
3. Jakarta shall have enough area for housing and greenspaces.

To achieve these three targets, the Government of Jakarta planned four large projects, a monorail, mass rapid transit (MRT), deep tunnel, and a giant sea wall in 2014 [54]. The deep tunnel is intended to store floodwaters during urban inundation. The giant sea wall was constructed to counter tsunamis. The Government of Jakarta also took actions to resolve water shortages, limited green areas, and water pollution. Non-governmental organizations and stakeholders have also become involved. The government of Jakarta has conducted several pilot projects, such as the Jakarta Coastal Defense Strategy (JCDS), Reconstruction of Pluit Basin, PAM Jaya’s Authority Transfer, and Jakarta Emergency Dredging Initiative (JEDI), which together approach an integrated sustainable urban development plan that can solve environmental challenges.

## 4. Water quality spatial trend analysis results

**Fig 3:**
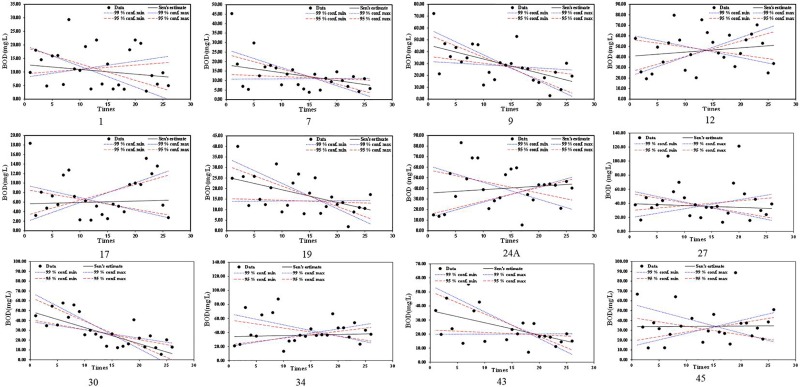
BOD trend analysis results with selected significant trend stations from 2008 to 2014(Three to five times sampling per year).

Trend analysis of BOD concentration from 44 observation stations showed most stations with decreasing trends, indicating that water quality in Jakarta has improved in recent years. However, some stations showed a significant increasing trend. Fig 3 shows the stations with their respective water quality trends. BOD of Station NO.1 shows a decreasing trend. High BOD values over 20 mg/l were observed, but recent BOD values have stabilized below 10 mg/l. Station NO. 9 shows BOD values with a significant decreasing trend, but extreme values over 70 mg/l in the first year. Station NO. 12 shows a significant increasing trend with an extreme value over 80 mg/l. All BOD values in Station NO. 12 are over 15 mg/l. BOD of Station NO. 17 had an increasing trend with most BOD values around 6 mg/l. Station NO. 19 shows a significant decreasing trend. BOD values range from under 17 mg/l to around 70 mg/l. The BOD value of Station NOs. 24, 34 and 45 had increasing trends. The distribution of BOD values comprises a large range from less than 5mg/l to over 80 mg/l. The BOD value of Station NO. 27 had an extreme value of 120 mg/l. Station NO. 30 shows a significant decreasing trend distributed closely around the trend line. Trend analysis of BOD values shows scattered values with some stations showing extreme values.

**Fig 4:**
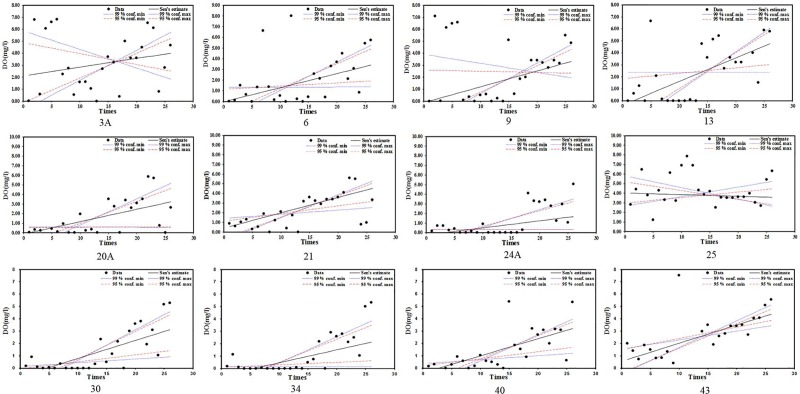
DO trend analysis results with selected significant trend stations from 2008 to 2014(Three to five times sampling per year).

DO generally shows an increasing trend (Fig 4). DO values for Station NOs. 9 and 13 had significant increasing trends during the recent 10 sampling events. Station NOs. 20A, 21 and 24 showed DO values with an increasing trend, with some low values during the recent 4 sampling events. Station NOs. 24A, 30 and 34 show increasing DO trends with many values in the early period close to zero. DO values for Station NO.25 show a decreasing trend. Station NO. 40 shows an increasing DO trend with values less than 6 mg/l. Station NO. 43 shows an increasing trend with recent improvements in DO.

**Fig 5:**
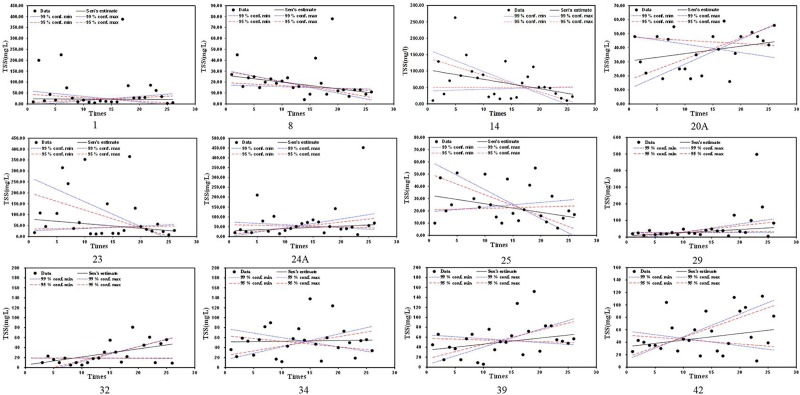
TSS trend analysis results with selected significant trend stations from 2008 to 2014(Three to five times sampling per year).

TSS trends included some significant trends (see Fig 5). TSS of Station NO. 1 shows a decreasing trend with an extreme value over 350 mg/l. Station NO.8 shows a significant decreasing trend in TSS with an extreme value under 50 mg/l. Station NO.14 shows a decreasing trend in TSS with an extreme value of 262 mg/l. TSS of Station NO. 20A shows a significant increasing trend with values in the range 10 to 60 mg/l. TSS trends for Station NOs. 23 and 25 have decreasing trends with quite different value ranges. Stations NO. 24A and 29 show increasing trends in TSS with most TSS values under 200 mg/l, although the recent period had extreme values around 500 mg/l. Stations NO. 32, 34, 39 and 42 show increasing trends in TSS, but the TSS trend for Station No. 34 is lower. TSS values of Station NO. 32 are under 81 mg/l. TSS values for Station NO. 42 are under 114 mg/l. TSS values for Stations NO. 34 and 39 are under 152 mg/l.

**Fig 6:**
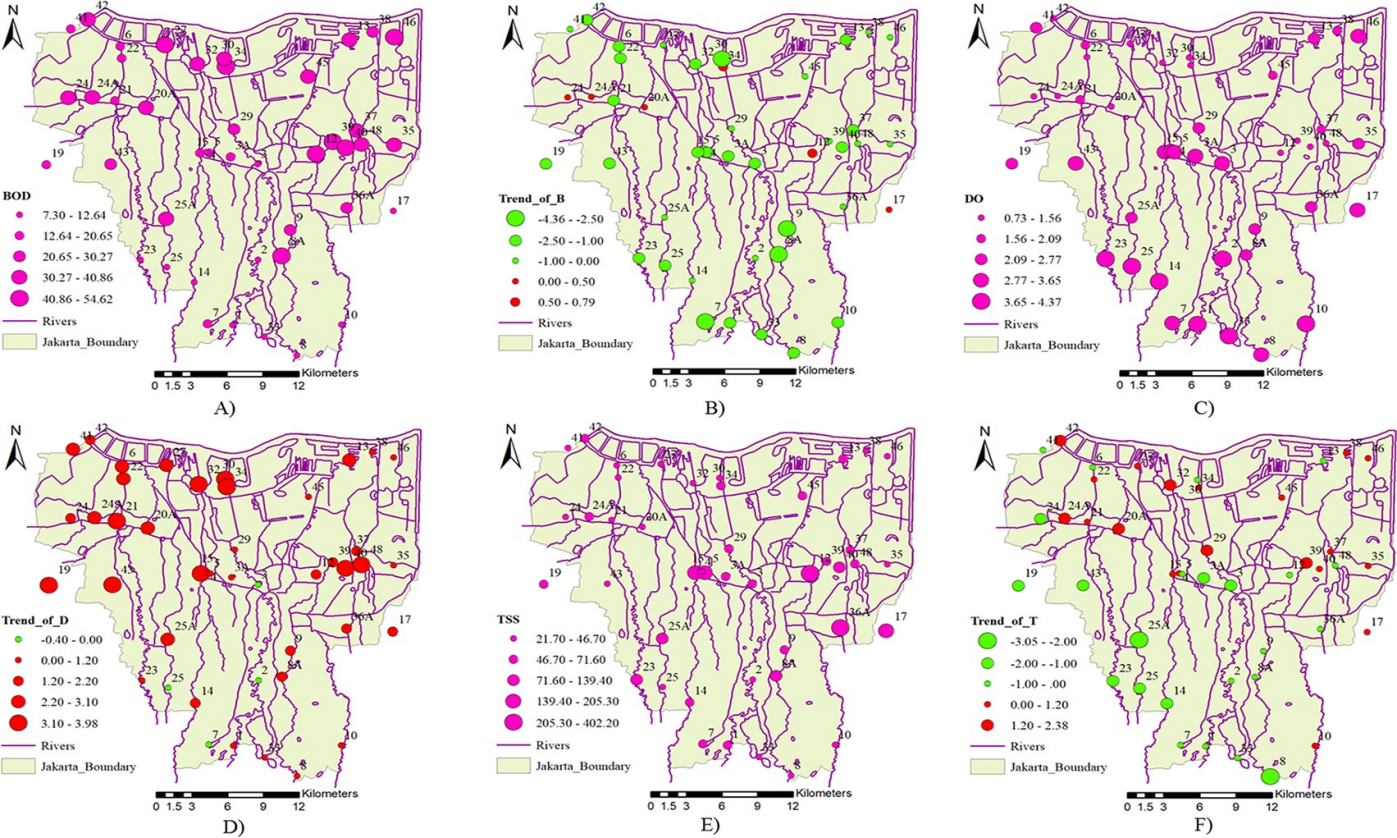
Spatial distribution of Jakarta water quality A) Average value of BOD; B) Trend Z value of BOD; C) Average value of DO; D) Trend Z value of DO; E) Average value of TSS; F) Trend Z value of TSS during the studying period. (The river network and boundary map are drawn by Dr. Apip; The average value and trend maps are drawn by Dr. Pingping Luo).

Fig 6 shows the low average values of BOD for the upper stream stations and the high average values of BOD for the downstream and middle stream of the eastern district of Jakarta. Most stations show significant decreasing trends in BOD, with the exceptions of NOs. 12, 17, 20A, 24, 24A and 30, which have small increasing trends in BOD in the middle and downstream. The average value of DO decreased significantly from upstream to downstream, with some stations in the central district showing high DO values. Most stations show significant increasing trends in DO, except for Stations NO. 2, 3, 7 and 25 which have decreasing trends in the upper and middle streams. This may be the result of rapid urbanization in Jakarta which increased housing development in the upper and middle streams. Most stations have low average values of TSS, but there are some stations located in the central and eastern districts that have high average values of TSS. This may result from construction and wastewater due to high population density and urbanization. TSS trends show a significant decreasing trend in the upper and middle streams. These trends show that TSS is connected to human activities.

**Fig 7:**
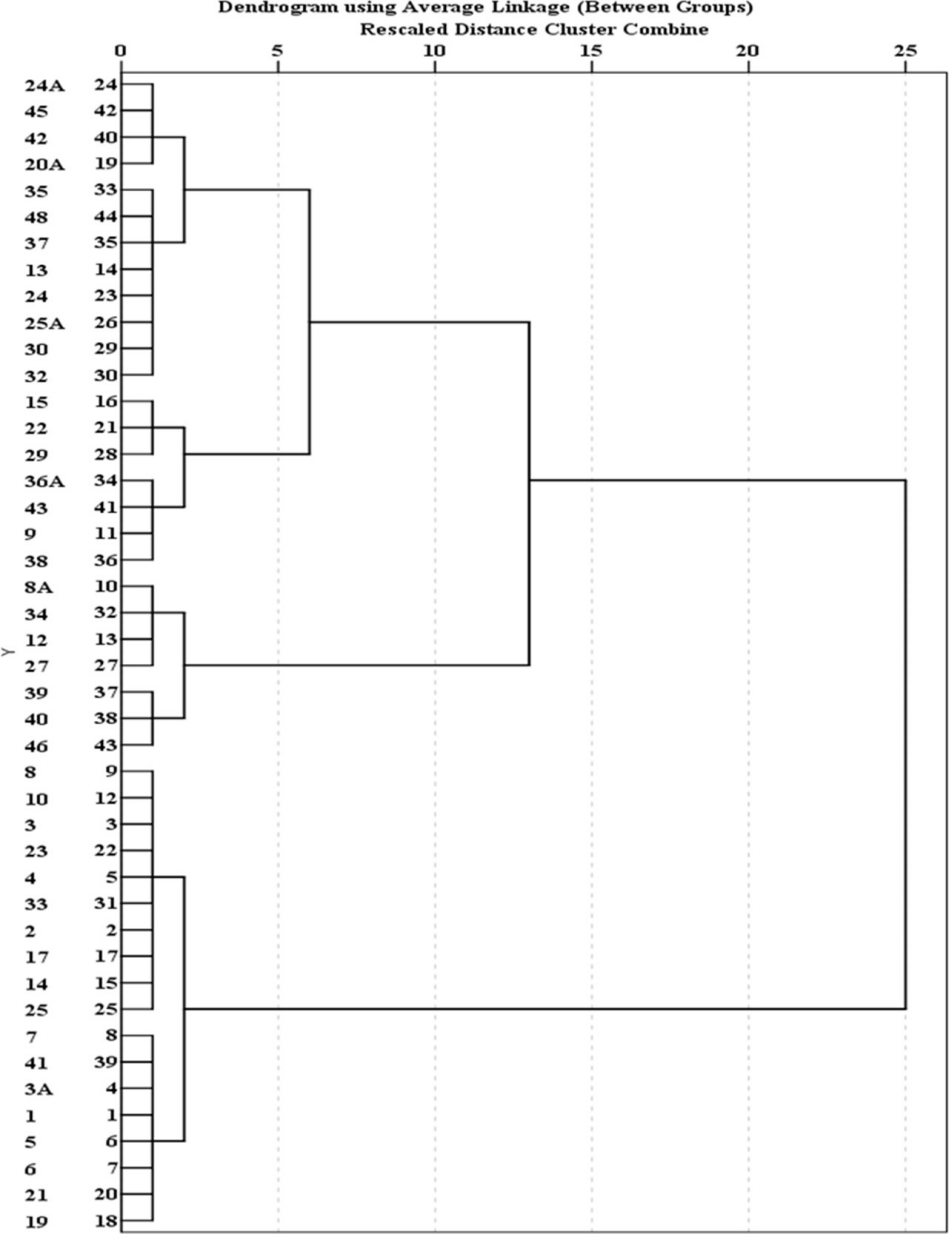
Dendogram showing spatial clustering of 44 water quality sampling stations of BOD.

**Fig 8:**
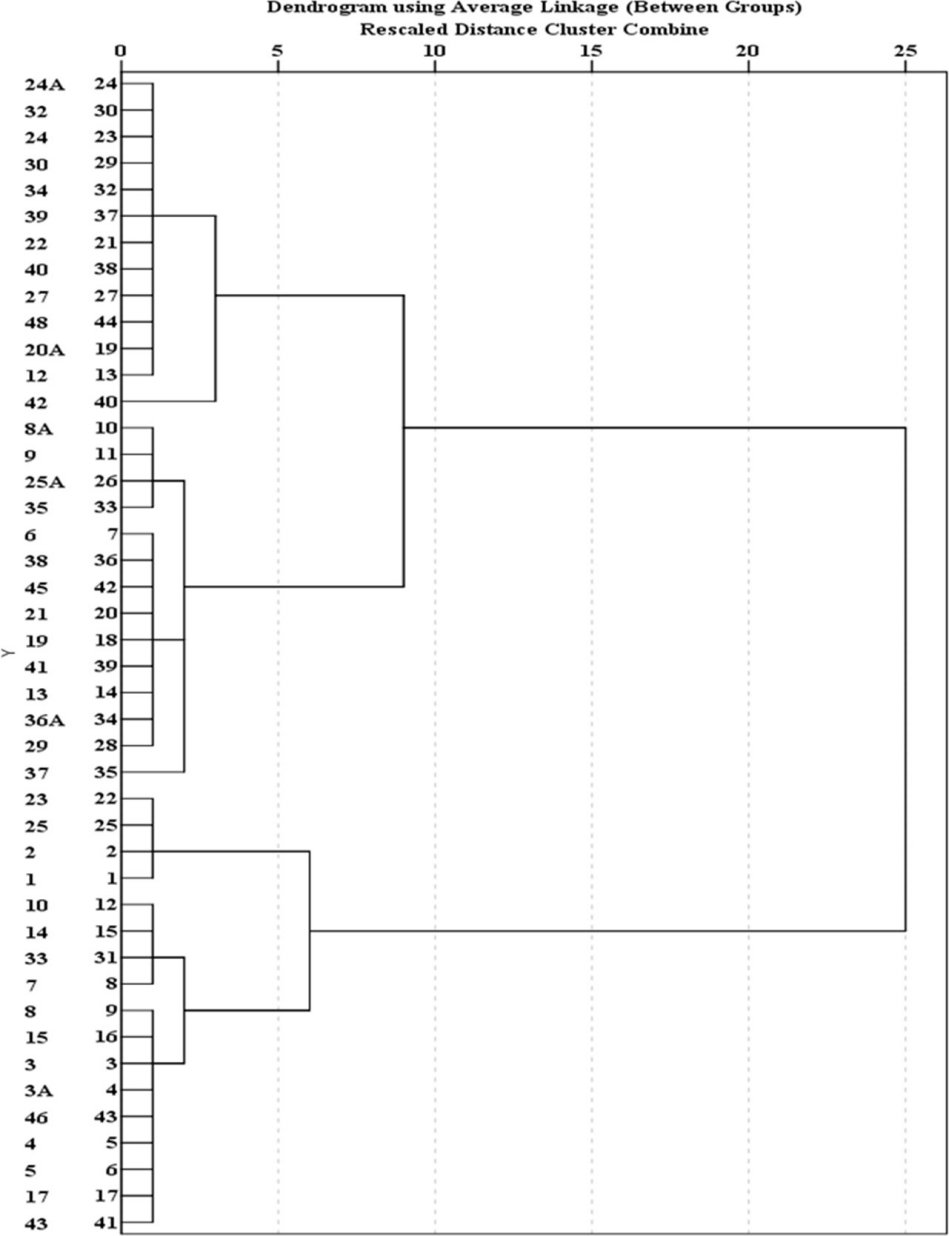
Dendogram showing spatial clustering of 44 water quality sampling stations of DO.

**Fig 9:**
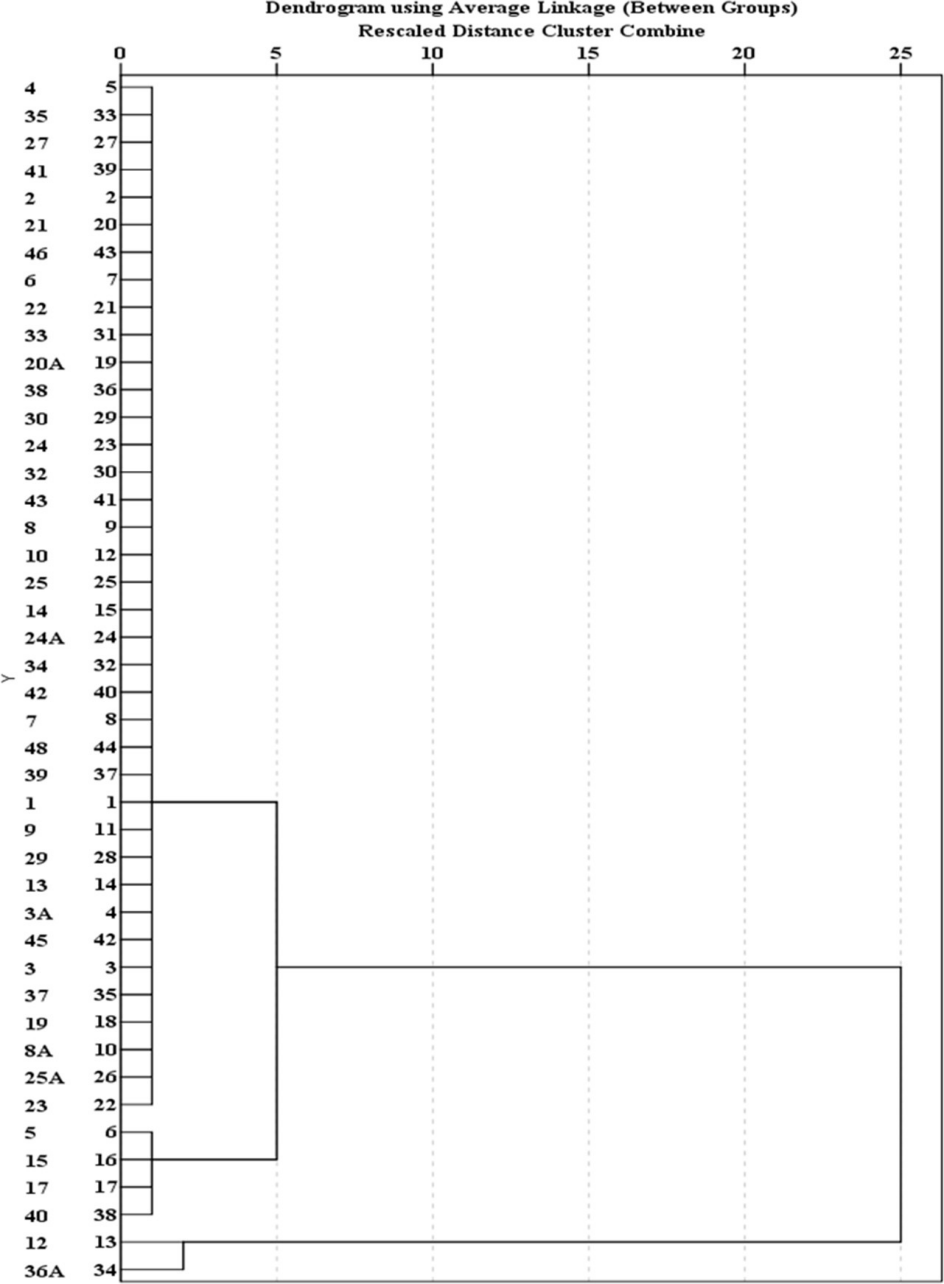
Dendogram showing spatial clustering of 44 water quality sampling stations of TSS.

Cluster analysis of 44 water quality sampling stations in Jakarta is shown in Figs 7, 8 and 9. Fig 7 groups 44 sites of BOD average concentration into three clusters. Sample sites 1, 2, 3, 3A, 4, 5, 6, 7, 8, 10, 14, 17, 19, 21, 23, 25, 33 and 41 are grouped into Cluster 1. Cluster 2 includes water quality stations 8A, 12, 27, 34, 39, 40, and 46. Other sites fall into Cluster 3. The results show that the concentration of BOD in Cluster 2 is highest and concentrated in the eastern region, followed by Cluster 1 with moderate pollution, and Cluster 3 with the lowest concentrations. Fig 8 groups 44 sites of DO average concentration into four clusters. Sample sites 12, 20A, 22, 24, 24A, 27, 30, 32, 34, 39, 40, 42 and 48 are grouped into Cluster 1. Cluster 2 includes the water quality stations 6, 8A, 9, 13, 19, 21, 25A, 29, 35, 36A, 37, 38, 41 and 45. Cluster 3 comprises water quality stations 1, 2, 23 and 25. Other sites fall into Cluster 4. Results show that the highest concentrations of DO are represented by Cluster 3 in the upstream, and Cluster 4 is characterized by severe pollution and is basically distributed in the middle, followed by Cluster 2 with moderate pollution. These sites are basically distributed in the middle and downstream. Cluster 1 has the lowest concentrations. Fig 9 groups 44 sites into three clusters. Sample sites 12 and 36A are grouped into Cluster 1. Cluster 2 includes water quality stations 5, 15, 17, and 40. Other sites fall into Cluster 3. The site classification is closely related to the concentration distribution, and the concentration order is: Cluster 3>2>1.

## 5. Recommendations for sustainable urban water environment management

Water pollution problems in Jakarta are serious due to the limited WWTP capacity, with 4% of housing in Jakarta covered by WWTP [55]. Indonesian Government Regulation No. 82 of 2001 regulated water pollution control and water quality management. According to the regulation, pollutant load capacity, water quality monitoring, and wastewater discharge determination should be identified and carried out by the national government. Data will help for building construction permits, spatial planning of water source management, wastewater discharge permits, and water quality identification.

The provincial and city governments are responsible for cross-city and cross-district water pollution problems and must provide a report of water pollution conditions every year to the Ministry of Environment. Two septic tank waste treatment installations with 300 m^3^ capacities are managed by the Jakarta Provincial Government (DKI Jakarta Province Government 2013). The population of Jakarta continued to increase from 2010 to 2014. Water use is also increasing, driving enormous challenges for WWTP development, planning and management. BOD and DO analysis show that water qaulity for most stations improved from 2008 to 2014. The improvement of water quality may come from water pollution control actions and the construction of WWTPs by the provincial and city government. However, there are some stations which still show poor water quality, possibly due to rising residential populations and lack of sewerage and WWTPs. It has been reported that only 4% of Jakarta has access to sewerage, with 96% or over 9.2 million people, withno wastewater management or treatment systems [56]. Most sewerage is disconnected and discharged directly to surface waters. Addressing environmental challenges requires complete sewerage connected to WWTP. The government of Jakarta is planning to build a USD 6.8 billion wastewater treatment system to cover Jakarta by 2022 [56].

Jakarta experiences many water-related problems due to lack of WWTP and sewerage connections. Rapid population growth is a major factor affecting water pollution problems. The construction of WWTP and sewerage cannot match the pace of population growth. Rapid urbanization is also driving overexploitation of groundwater and land subsidence in Jakarta adding to the many water sources facing stress in the city. Water supply in Jakarta is achieved via the combined sources of piped water, groundwater, bottled water and recycled water [57]. Water access must be made more equitable and more environmentally sustainable by developing robust plans for water service delivery and inclusive development in the city. Over 90% of domestic wastewater is managed via on-site systems (septic tank and septage treatment) with low treatment quality. Existing facilities often fail due to high cost of investment, operations and maintenance [58]. Construction of a comprehensive sewage system is essential to connect to WWTP in urban areas to control water pollution problems. Ancillary problems include rural wastewater collection, and solid waste management. Sustainable urban water environments require managing flood risk exacerbated by land subsidence due to groundwater overdraft [59]. Urban inundation can drive solid waste and polluted water into surface waters exacerbating pollution problems [60]. Limiting groundwater overdraft is essential to addressing flood risk as is managing groundwater recharge, construction of pump stations and underground water storage. Government has the responsibility to regulate the private sector and build sewage systems connected to WWTP. Collaboration between citizens and government is a powerful way to improve water quality.

Best practice dictates that improving water quality requires wastewater collection and connection to sewerage and ultimately to the WWTP. WWTPs must be designed considering population growth and urban development. Water pollution controls must be implemented to eliminate overexploitation of ground water, prevent flood disasters, and use ecological measures to clean the urban waters according to comprehensive urban development plans. Improved water quality and water environment can lend value to Jakarta and improve the aesthetic quality of the city as well as living conditions. Clean river water confers human health, tourism development, real estate value, and commerce, in addition to happiness. The limitation of this paper is that the sampling times are not long, and the data quantity is insufficient. The water quality parameters are also insufficient, and other factors such as COD, TP, TN, NH_3_-N should also be fully considered. Future research should consider (1) the effect of flooding on water quality; (2) groundwater pollution; and (3) field surveys on pollution sources.

## 6. Conclusions

This study focuses on the relationship between urbanization and water stress as well as trend analysis of BOD, TSS and DO. Jakarta has recently seen rapid population growth, water shortages, flood risk, and land subsidence caused by groundwater overdraft. These problems are especially acute for Jakarta which lacks wastewater treatment plants and sewerage. BOD and TSS in most stations show a decreasing trend. Some stations in the downstream show significant increasing trends. DO in most stations shows an increasing trend. However, DO values in most stations are lower than 5 mg/l. Although water quality in Jakarta has generally improved in recent years, there is still a great deal of work to do. Sufficient WWTP capacity is necessary to deliver high quality effluent to rivers and streams. Wastewater treatment plants should consider population growth and urban development in the present and future. For rural areas, collecting wastewater and solid waste can be effective to stop river water pollution. Limiting groundwater overdraft can effectively stop land subsidence and mitigate flooding. Cooperation between government, industry and the public is necessary to address water quality and environmental challenges. Results from this study provide valuable information that resource managers can use to create sustainable water environments in Jakarta and beyond.

## Supporting information

S1 File Data new. The concentration values of DO, BOD and TSS at the 44 sampling points from 2008 to 2014.
(DOCX)
