# Supplementary material for: Water quality trend assessment in Jakarta: A rapidly growing Asian megacity
Source: PLoS One. 2019 Jul 11;14(7):e0219009. doi: 10.1371/journal.pone.0219009 (PMC6623954; doi:10.1371/journal.pone.0219009)
Supplement: S1 File — The concentration values of DO, BOD and TSS at the 44 sampling points from 2008 to 2014. (DOCX) [file pone.0219009.s001.docx]

|  | **Date**  **Sites** | **2008/8/1** | **2008/10/1** | **2008/11/1** | **2009/4/1** | **2009/7/1** | **2009/10/1** | **2010/3/1** | **2010/5/1** | **2010/8/1** | **2011/4/1** | **2011/7/1** | **2011/9/1** | **2011/10/1** | **2012/5/1** | **2012/6/1** | **2012/9/1** | **2012/10/1** | **2012/11/1** | **2013/2/1** | **2013/6/1** | **2013/10/1** | **2014/2/1** | **2014/5/1** | **2014/6/1** | **2014/9/1** | **2014/10/1** |
| --- | --- | --- | --- | --- | --- | --- | --- | --- | --- | --- | --- | --- | --- | --- | --- | --- | --- | --- | --- | --- | --- | --- | --- | --- | --- | --- | --- |
| **DO** | 1 | 3.51 | 4.38 | 3.65 | 5.55 | 2.3 | 4.44 | 6.66 | 4.62 | 2.46 | 8.1 | 6.1 | 1.56 | 3.23 | 4 | 3.8 | 2.41 | 2.72 | 3.2 | 3.4 | 3.7 | 4 | 3.97 | 3.91 | 3.7 | 5.69 | 6.51 |
|  | 2 | 2.71 | 3.03 | 3.41 | 4.24 | 2.69 | 4.44 | 8.56 | 5.26 | 7.73 | 4.14 | 4.68 | 7.5 | 6.4 | 4.6 | 4 | 3.51 | 0.93 | NA | 3.6 | 3.7 | 3.2 | 3.95 | 3.98 | 5.27 | 5.6 | 1.94 |
|  | 3 | 0.14 | 6.82 | 1.54 | 6.335 | 6.5 | 6.79 | 4.6 | 2.39 | 1.54 | 1.09 | 0.5 | 2 | 1 | 0.46 | 2.9 | 3.57 | 0.71 | 5.8 | 3.6 | 3.6 | 3.8 | 3.2 | 3.45 | 3.8 | 5.43 | 0.32 |
|  | 3A | 0.02 | 6.8 | 0.57 | 6.055 | 6.59 | 6.82 | 2.25 | 2.74 | 0.54 | 1.59 | 1.61 | 1.04 | 0 | 2.7 | 3.7 | 3.24 | 0.38 | 5 | 3.6 | 3.6 | 4.5 | 6.53 | 6.1 | 0.81 | 2.8 | 4.67 |
|  | 4 | 0.04 | 6.6 | 0.82 | 6.375 | 5.84 | 6.68 | 3.42 | 2.14 | 0 | 2.42 | 0.82 | 0.13 | 0.64 | 3.41 | 4 | 1.2 | 4.19 | 3.4 | 3.6 | 3.6 | 4.7 | 6.32 | 5.98 | 1.36 | 3.26 | 4.6 |
|  | 5 | 0 | 6.8 | 0.9 | 5.99 | 6.54 | 6.61 | 3.44 | 2.02 | 0 | 3.18 | 0.92 | 0.1 | 0 | 3.31 | 4.1 | 3.18 | 3.18 | 3.4 | 3.5 | 3.6 | 3.7 | 7.03 | 6.53 | 1.3 | 0.51 | 2.55 |
|  | 6 | 0.03 | 0.13 | 1.52 | 0.66 | 0 | 1.34 | 6.62 | 1.37 | 0.15 | 0.55 | 0 | 8 | 0.24 | 0 | 0.43 | 2.6 | 2.14 | 0.4 | 3.3 | 3.7 | 4.5 | 2.13 | 3.09 | 0.85 | 5.43 | 5.74 |
|  | 7 | 1.67 | 4.4 | 2.97 | 3.965 | 5.29 | 4.78 | 0.89 | 7.25 | 4.42 | 4.46 | 5.78 | 1.1 | 1.11 | 3.84 | 3.6 | 1.48 | 4.22 | 2 | 3.5 | 3.6 | 3.2 | 3.14 | 3.36 | 3.21 | 4.6 | 6.35 |
|  | 8 | 7.11 | 0.95 | 6.48 | 5.86 | 0.89 | 6.41 | 1.25 | 1.82 | 2.77 | 3.96 | 3.6 | 2.87 | 1 | 3.28 | 3.1 | 1.06 | 2.51 | 3 | 3.4 | 3.6 | 4.2 | 2.88 | 2.5 | 1.77 | 5.86 | 5.92 |
|  | 8A | 7.43 | 0 | 6.52 | 6.25 | 1.16 | 6.4 |  | 0.43 | 0 | 0 | 0 | 0 | 0.23 | 2.63 | 3.2 | 0.12 | 2.69 | 2.7 | 3.2 | 3.4 | 3.8 | 3.3 | 3.23 | 1.46 | 5.38 | 4.86 |
|  | 9 | 0 | 7.1 | 0.02 | 6.135 | 6.48 | 6.58 | 0.08 | 0.35 | 0 | 0.5 | 0.57 | 0 | 0.23 | 0 | 5.1 | 0.6 | 1.87 | 2 | 3.4 | 3.4 | 3.2 | 2.8 | 3.4 | 3.15 | 5.49 | 4.85 |
|  | 10 | 7.51 | 0.11 | 6.44 | 6.015 | 0.5 | 6.35 | 0.5 | 4.07 | 3.71 | 5.51 | 4.29 | 1.1 | 1.15 | 2.88 | 3.6 | 2.4 | 2.62 | 3 | 3.6 | 3.5 | 4.3 | 3.38 | 7.03 | 3.22 | 5.9 | 6.04 |
|  | 12 | 0 | 0.7 | 1.3 | 0.05 | 6.25 | 2 | 0 | 0 | 2.21 | 0 | 0 | 0 | 0 | 0.72 | 0.2 | 2.94 | 0.68 | 5.6 | 3.2 | 2.8 | 2.6 | 0.8 | 2.64 | 2.62 | 2.77 | 0.28 |
|  | 13 | 0 | 0.6 | 1.24 | 0 | 6.64 | 2.07 | 0.15 | 0 | 0 | 0 | 0 | 0.1 | 0 | 4.76 | 3.6 | 5.12 | 5.42 | 2.7 | 3.6 | 3.2 | 3.2 | 6.85 | 3.99 | 1.52 | 5.89 | 5.79 |
|  | 14 | 2.45 | 2.99 | 2.75 | 3.46 | 2.53 | 2.94 | 4.46 | 7.34 | 3.14 | 5.53 | 3.8 | 4.2 | 3 | 4.15 | 4 | 2.63 | 3.69 | 3.3 | 3.5 | 3.6 | 3.5 | 4.03 | 4.39 | 2.47 | 5.81 | 6.33 |
|  | 15 | 0.15 | 1.2 | 0.08 | 0 | 0.09 | 0 | 2.38 | 1.86 | 1.08 | 2.09 | 2.6 | 0 | 0 | 1.01 | 1 | 0 | 57.5 | 2.4 | 3.3 | 3.6 | 3.7 | 3.05 | 6.16 | 6.04 | 3.54 | 2.55 |
|  | 17 | 2.29 | 3.11 | 4.3 | 3.75 | 1.79 | 2.95 | 3.93 | 4.82 | 3.24 | 2.7 | 0.46 | 0.9 | 1.96 | 0 | 6 | 4.35 | 1.89 | 1.1 | 3.5 | 3.4 | 3.5 | 4.42 | 4.61 | 4.35 | 2.75 | 5.75 |
|  | 19 | 0.05 | 0.5 | 1.47 | 1.15 | 0.32 | 2.95 | 3.72 | 1.59 | 1.3 | 2.24 | 0.78 | 0.06 | 0 | 3.16 | 3.7 | 1.81 | 0.93 | 3.4 | 3.4 | 3.4 | 3.7 | 6.06 | 3.13 | 6.02 | 3.73 | 5.78 |
|  | 20A | 0.05 | 0.33 | 0.22 |  | 0.42 | 0.11 | 0.93 | 0 | 0 | 1.94 | 0.24 | 0.36 | 0 | NA | 3.5 | 2.74 | 0 | 3.4 | 2.6 | 3.1 | 3.5 | 5.86 | 5.7 | 0.76 | 0 | 2.61 |
|  | 21 | 0.87 | 0.6 | 1.02 | 1.31 | 0.31 | 0.55 | 1.9 | 0 | 1.2 | 2.09 | 0.38 | 1.74 | 0 | 3.17 | 3.6 | 3.22 | 2.91 | 3.4 | 3.4 | 3.6 | 4.1 | 5.59 | 5.49 | 0.78 | 0.98 | 3.32 |
|  | 22 | 0.74 | 1.93 | 0.01 |  | 1.54 | 0 | 0.23 | 0.63 | 0 | 0.44 | 0 | 0 | 0.45 | 0.36 | 0.4 | 0.93 | 1.94 | 0 | 2.8 | 3.6 | 4.5 | 2.08 | 3.38 | 0.72 | 5.13 | 2.33 |
|  | 23 | 4.36 | 2.62 | 4.78 | 3.74 | 3.18 | 4.42 | 3.78 | 6 | 4.1 | 6.29 | 5.3 | 4.6 | 4.7 | 4.36 | 5.3 | 2.88 | 4.38 | 3.5 | 3.4 | 3.6 | 3.2 | 5.05 | 4.27 | 2.98 | 6.17 | 6.3 |
|  | 24 | 0.88 | 0.81 | 1.17 | 1.105 | 0.22 | 0 | 0 | 0 | 1.16 | 1.08 | 2.8 | 0 | 0 | 0.5 | 0 | 0 | 0 | 0.8 | 2.8 | 3.4 | 3.6 | 2.7 | 3.09 | 3.01 | 0.99 | 0.58 |
|  | 24A | 0.17 | 0.71 | 0.72 | 0.25 | 0.42 | 0 | 0 | 0.18 | 0 | 0.89 | 0 | 0 | 0 | 0 | 0 | 0 | 0.3 | 4.1 | 3.3 | 3.2 | 3.4 | 2.78 | 1.25 | 2.97 | 1.07 | 5.06 |
|  | 25 | 2.82 | 4.41 | 6.45 | 3.785 | 1.22 | 4.29 | 3.3 | 6.13 | 3.2 | 6.88 | 7.86 | 6.9 | 4.3 | 3.86 | 4.2 | 2.51 | 3.56 | 3.5 | 3.5 | 3.6 | 3.6 | 3.97 | 3.01 | 2.7 | 5.41 | 6.32 |
|  | 25A | 0.44 | 2.73 | 6.36 | 2.82 | 0.34 | 3 | 1.52 | 4.01 | 1.32 | 3.84 | 1.6 | 2.8 | 0 | 2.9 | 3.2 | 0.99 | 2.49 | 2.9 | 2.6 | 3.6 | 3.7 | 2.77 | 4.16 | 3.62 | 4.16 | 5.47 |
|  | 27 | 0 | 1.8 | 6.43 | 1.225 | 0 | 1.16 | 0.09 | 0 | 0 | 0.59 | 0 | 0 | 0.77 | 0 | 0.4 | 0.76 | 1.89 | 0 | 2.6 | 2.6 | 3 | 1.43 | 3.03 | 0.95 | 5.07 | 4.4 |
|  | 29 | 0.59 | 6.7 | 0.31 | 6.26 | 6.18 | 1.16 | 1.46 | 0.53 | 1.02 | 0 | 0 | 1.2 | 0 | 0.43 | 0 | 3.19 | 0.66 | 5.7 | 2.7 | 3.5 | 4.5 | 1.1 | 3.71 | 3.28 | 5.53 | 0.78 |
|  | 30 | 0.17 | 0.91 | 0.11 | 0 | 0.06 | 0 | 0.35 | 0 | 0 | 0 | 0 | 0 | 0.34 | 2.33 | 0.5 | 1.15 | 2.15 | 0 | 3 | 3.6 | 3.8 | 1.93 | 3.08 | 1.03 | 5.16 | 5.27 |
|  | 32 | NA | NA | 0.09 | 0 | 0 | 0 | 0.5 | 0.56 | 0 | 0 | 0 | 0 | 0.48 | 0.96 | 0.4 | 1.41 | 1.96 | 0 | 2.9 | 3.4 | 4.3 | 1.57 | 2.24 | 1.15 | 4.86 | 4.14 |
|  | 33 | 1.51 | 4.11 | 3.16 | 3.945 | 1.8 | 2.63 | 6.39 | 5.46 | 4.97 | 8.33 | 5.75 | 2.1 | 1.3 | 3.9 | 3.7 | 2.3 | 2.61 | 2.1 | 2.5 | 3.6 | 4.4 | 3.6 | 3.73 | 2.34 | 5.66 | 5.61 |
|  | 34 | 0.18 | 1.13 | 0.1 | 0 | 0 | 0 | 0.06 | 0 | 0 | 0 | 0 | 0 | 0.08 | 0 | 0.5 | 0.75 | 2.17 | 0 | 2.9 | 2.6 | 2.8 | 2.14 | 2.5 | 1.04 | 5 | 5.33 |
|  | 35 | 0.28 | 6.5 | 6.2 | 1.605 | NA | 0.96 | 0.12 | 0 | 0 | 1.39 | 0 | 0 | 0 | 4.62 | 3.9 | 5.71 | 6.11 | 2.5 | 3.3 | 3.3 | 3.3 | 6.92 | 5.11 | 2.8 | 2.64 | 2.7 |
|  | 36A | 0.2 | 2.6 | 4.6 | 1.08 | 1.05 | 2.35 | 0.88 | 0.64 | 0 | 3.92 | 0.36 | 0.59 | 3.08 | 0 | 7.3 | 3.15 | 1.76 | 0.8 | 3.3 | 2.8 | 3.2 | 1.93 | 4.02 | 4.25 | 1.66 | 6.05 |
|  | 37 | 0.31 | 6.2 | 4.16 | 0 | 0 | 1.21 | 0.23 | 0 | 0 | 0.9 | 0 | 0 | 0.05 | 0 | 5.3 | 0.95 | 1.07 | 0.9 | 3.4 | 3.4 | 3.2 | 1.65 | 3.51 | 3.48 | 5.55 | 0.44 |
|  | 38 | 0.68 | 6.92 | 4.44 | 0 | NA | 0.72 | 0.13 | 0 | 0 | 0.85 | 0 | 0 | 0 | 4.96 | 3.4 | 5.15 | 4.2 | 2.5 | 3.2 | 3.4 | NA | 6.6 | 5 | 1.24 | 0.28 | 0 |
|  | 39 | 0.11 | 0.51 | 0.05 | 0 | 0.57 | 0.51 | 0 | 0 | 0 | 1.17 | 0 | 0 | 0 | 0.46 | 0 | 2.99 | 0.62 | 5.6 | 2.8 | 3.1 | 3.2 | 0 | 0 | 3.16 | 1.95 | 0 |
|  | 40 | 0.16 | 0.3 | NA | 0 | 0.29 | 0.93 | 0.59 | 0 | 0.18 | 1.04 | 0.59 | 0.5 | 0.29 | 0 | 5.4 | 1.87 | 1.54 | 0.9 | 3.2 | 2.7 | 3.1 | 2 | 3.17 | 3.1 | 0.63 | 5.35 |
|  | 41 | 3.69 | 1.24 | 1.44 | 1.5 | 0.53 | 0.66 | 1.44 | 2.42 | 1.49 | 2.4 | 2.8 | 4 | 3.1 | 2.24 | 1.1 | 0 | 0.31 | 2.1 | 3 | 3.6 | 3.7 | 4.12 | 3.15 | 5.09 | 2.91 | 3.41 |
|  | 42 | 0.09 | 0.1 | 0.47 | 0 | 0 | 0.05 | 0 | 0 | 0 | 0 | 1.3 | 0 | 0 | 0 | 0 | 0 | 0.43 | 0 | 3.4 | 2.8 | 3.2 | 3.5 | 0.11 | 1.87 | 1.02 | 0 |
|  | 43 | 2 | 1.4 | 0.73 | 1.85 | 1.5 | 0.82 | 0.83 | 1.33 | 0.41 | 7.5 | NA | NA | NA | 2.98 | 3.5 | 1.91 | 2.59 | 2.8 | 3.4 | 3.4 | 3.5 | 2.7 | 4.05 | 4.08 | 5.1 | 5.56 |
|  | 45 | 0 | 0.63 | 1.57 | 0.09 | 6.18 | 1.96 | 0 | 0 | 0 | 0 | 0 | 0 | 0 | 28.6 | 0 | 3.09 | 0.62 | 5.4 | 2.5 | 2.7 | 2.8 | 0 | 0 | 2.61 | 0.51 | 0 |
|  | 46 | 2.71 | 6.30 | 4.71 | 1.36 | 0.51 | 1.51 | 3.96 | 1.83 | 4.37 | 3.18 | 5.20 | 1.45 | 1.10 | 5.64 | 4.60 | 4.20 | 5.06 | 2.40 | 3.60 | 2.40 | 2.40 | 6.79 | 4.06 | 3.57 | 3.10 | 1.27 |
|  | 48 | 0.13 | 0.62 | 0.60 | 0 | 0.13 | 2.44 | 0.62 | 0.53 | 0.01 | 1.25 | 0.45 | 0 | 0.29 | NA | 5.20 | 0.67 | 1.59 | 1.00 | 3.20 | 3.40 | 3.20 | 1.82 | 3.16 | 3.48 | 2.55 | 1.47 |
| **BOD** | 1 | 9.85 | 18.05 | 14.55 | 4.85 | 15.95 | 16.10 | 5.40 | 29.35 | 11.15 | 10.60 | 19.39 | 3.75 | 21.80 | 5.58 | 12.95 | 3.76 | 5.24 | 3.67 | 18.20 | 21.80 | 20.60 | 2.97 | 8.69 | 5.54 | 9.73 | 4.98 |
|  | 2 | 6.25 | 4.86 | 12.95 | 6.60 | 9.20 | 14.55 | 4.90 | 6.80 | 7.21 | 2.55 | 9.34 | 6.18 | 7.62 | 6.20 | 11.23 | 6.29 | 6.14 | NA | 6.00 | 3.80 | 8.00 | 4.37 | 7.89 | 3.55 | 10.02 | 8.54 |
|  | 3 | 17.73 | 15.55 | 3.80 | 4.70 | 15.40 | 15.45 | 6.35 | 17.15 | 9.30 | 5.15 | 22.58 | 8.95 | 11.42 | 15.51 | 6.27 | 8.76 | 11.52 | 4.78 | 11.80 | 5.60 | 7.40 | 3.40 | 12.70 | 5.23 | 9.93 | 9.81 |
|  | 3A | 29.85 | 42.30 | 9.20 | 6.25 | 23.80 | 30.15 | 5.60 | 12.55 | 23.60 | 3.05 | 20.78 | 7.01 | 8.56 | 5.27 | 4.86 | 13.13 | 13.74 | 9.03 | 12.10 | 6.20 | 5.70 | 8.91 | 6.68 | 7.02 | 9.71 | 9.20 |
|  | 4 | 27.60 | 10.25 | 7.80 | 8.25 | 13.10 | 10.40 | 7.20 | 9.90 | 16.00 | 7.30 | 22.22 | 14.29 | 12.94 | 6.00 | 6.98 | 12.52 | 7.31 | 9.02 | 12.40 | 7.50 | 8.10 | 1.93 | 11.70 | 4.55 | 10.02 | 15.52 |
|  | 5 | 22.90 | 10.05 | 6.00 | 6.85 | 41.85 | 10.85 | 15.30 | 14.20 | 84.65 | 6.84 | 11.05 | 10.78 | 11.80 | 19.32 | 9.71 | 14.42 | 14.34 | 14.48 | 12.90 | 9.60 | 8.60 | 3.96 | 7.89 | 4.75 | 6.38 | 18.29 |
|  | 6 | 32.43 | 18.33 | 8.00 | 7.40 | 48.75 | 11.70 | 15.00 | 18.90 | 16.00 | 5.70 | 16.40 | 24.32 | 22.88 | 8.02 | 25.60 | 14.63 | 12.98 | 9.57 | 24.30 | 9.40 | 10.40 | 3.96 | 5.58 | 4.02 | 14.60 | 12.80 |
|  | 7 | 45.30 | 18.70 | 6.95 | 5.35 | 29.80 | 12.50 | 17.20 | 18.00 | 16.65 | 7.75 | 13.39 | 15.70 | 7.86 | 5.58 | 3.84 | 13.33 | 4.97 | 11.19 | 9.40 | 14.60 | 10.00 | 6.89 | 12.10 | 4.24 | 10.98 | 5.80 |
|  | 8 | 15.45 | 8.10 | 18.04 | 7.20 | 8.75 | 14.73 | 10.20 | 9.95 | 9.10 | 10.20 | 11.80 | 13.48 | 13.24 | 4.96 | 9.21 | 20.98 | 6.75 | 8.95 | 19.30 | 9.20 | 7.50 | 1.46 | 10.90 | 3.19 | 5.30 | 8.86 |
|  | 8A | 124.20 | 151.50 | 26.85 | 24.10 | 87.25 | 107.70 | 59.40 | 45.20 | 42.30 | 10.40 | 70.60 | 23.20 | 71.00 | 19.32 | 32.68 | 42.42 | 11.52 | 6.32 | 35.50 | 24.90 | 25.20 | 11.67 | 12.10 | 5.08 | 20.03 | 46.31 |
|  | 9 | 72.20 | 21.35 | 46.77 | 35.85 | 43.55 | 31.40 | 34.80 | 46.50 | 45.90 | 11.90 | 22.60 | 16.40 | 30.70 | 28.68 | 30.02 | 52.87 | 26.33 | 25.55 | 16.00 | 14.10 | 18.00 | 2.94 | 22.66 | 8.02 | 30.25 | 19.43 |
|  | 10 | 24.20 | 10.20 | 15.00 | 4.05 | 16.30 | 13.10 | 6.25 | 7.70 | 8.20 | 11.30 | 4.60 | 10.63 | 8.06 | 3.60 | 4.55 | 31.72 | 4.22 | 11.49 | 5.30 | 19.00 | 19.70 | 4.89 | 3.96 | 5.23 | 3.04 | 8.62 |
|  | 12 | 57.60 | 25.80 | 19.35 | 23.80 | 49.35 | 35.20 | 53.40 | 79.60 | 56.05 | 27.40 | 42.20 | 20.30 | 75.30 | 63.16 | 53.93 | 43.78 | 39.70 | 60.65 | 30.90 | 43.30 | 56.20 | 61.71 | 70.46 | 53.05 | 24.88 | 33.79 |
|  | 13 | 25.10 | 39.75 | 18.90 | 31.10 | 49.35 | 21.65 | 66.15 | 62.70 | 60.00 | 40.40 | 24.62 | 20.80 | 31.52 | 47.05 | 20.94 | 23.80 | 23.52 | 15.77 | 8.70 | 30.80 | 30.90 | 19.12 | 28.34 | 23.14 | 29.23 | 21.60 |
|  | 14 | 4.20 | 10.10 | 5.90 | 4.60 | 19.70 | 6.40 | 16.70 | 10.90 | 17.05 | 8.75 | 4.20 | 8.22 | 8.52 | 3.71 | 3.05 | 3.76 | 8.20 | 3.90 | 9.40 | 10.10 | 10.30 | 7.60 | 8.69 | 4.75 | 5.78 | 5.35 |
|  | 15 | 31.20 | 20.05 | 21.40 | 10.85 | 76.65 | 10.70 | 14.30 | 13.10 | 28.20 | 10.80 | 6.90 | 33.40 | 19.90 | 23.20 | 15.14 | 18.11 | 12.17 | 9.17 | 29.00 | 8.40 | 8.60 | 7.84 | 10.90 | 9.71 | 33.12 | 8.86 |
|  | 17 | 18.35 | 3.21 | 8.10 | 4.75 | 7.30 | 5.50 | 11.69 | 12.75 | 7.20 | 2.28 | 6.27 | 2.22 | 5.16 | 3.40 | 2.53 | 5.57 | 5.14 | 3.97 | 9.70 | 10.00 | 9.70 | 15.19 | 11.97 | 13.58 | 5.36 | 2.75 |
|  | 19 | 24.90 | 40.00 | 25.70 | 11.95 | 25.80 | 14.90 | 12.40 | 20.40 | 31.70 | 8.95 | 12.07 | 22.61 | 26.90 | 8.08 | 17.87 | 25.10 | 8.24 | 11.47 | 16.00 | 12.50 | 13.40 | 1.93 | 8.89 | 11.01 | 10.59 | 17.16 |
|  | 20A | 40.35 | 45.60 | 59.90 | NA | 59.40 | 19.00 | 36.00 | 38.15 | 64.26 | 3.10 | 10.81 | 11.20 | 29.70 | NA | 46.17 | 24.04 | 26.86 | 14.40 | 59.20 | 35.80 | 36.20 | 55.27 | 46.39 | 26.36 | 81.08 | 39.71 |
|  | 21 | 24.30 | 15.10 | 4.45 | 12.20 | 14.70 | 8.80 | 9.25 | 21.15 | 20.40 | 8.75 | 11.14 | 12.58 | 15.28 | 5.89 | 48.53 | 19.42 | 14.63 | 5.96 | 45.40 | 17.60 | 7.50 | 6.89 | 4.28 | 10.18 | 8.83 | 6.31 |
|  | 22 | 24.35 | 30.15 | 16.50 | NA | 33.00 | 15.10 | 18.40 | 24.00 | 16.90 | 15.55 | 13.09 | 22.22 | 19.33 | 13.43 | 31.33 | 20.17 | 22.47 | 15.97 | 46.40 | 12.50 | 13.40 | 7.84 | 11.63 | 11.19 | 15.21 | 27.92 |
|  | 23 | 5.40 | 7.30 | 6.65 | 19.60 | 12.80 | 12.20 | 12.15 | 13.10 | 11.10 | 10.90 | 11.29 | 10.50 | 15.28 | 8.02 | 9.21 | 3.76 | 6.66 | 19.30 | 14.10 | 8.70 | 16.10 | 2.94 | 6.48 | 3.40 | 2.34 | 5.20 |
|  | 24 | 19.60 | 24.10 | 21.70 | 29.90 | 29.00 | 50.00 | 20.65 | 59.30 | 58.20 | 22.55 | 22.37 | 36.10 | 30.90 | 12.18 | 26.61 | 40.33 | 21.48 | 30.22 | 69.80 | 15.10 | 15.40 | 44.15 | 16.31 | 28.49 | 21.82 | 66.86 |
|  | 24A | 14.95 | 13.55 | 15.15 | 54.05 | 32.40 | 83.20 | 49.00 | 68.75 | 68.75 | 38.80 | 20.87 | 28.30 | 31.10 | 52.94 | 58.31 | 59.47 | 5.44 | 34.12 | 29.10 | 43.40 | 43.50 | 44.15 | 43.05 | 21.15 | 46.57 | 40.27 |
|  | 25 | 4.65 | 17.08 | 14.10 | 6.30 | 14.10 | 14.75 | 13.10 | 7.95 | 12.80 | 15.65 | 4.59 | 11.11 | 5.43 | 5.58 | 6.27 | 5.84 | 4.40 | 10.11 | 6.20 | 10.00 | 10.20 | 3.35 | 4.38 | 7.91 | 7.58 | 8.92 |
|  | 25A | 19.20 | 329.5 | 10.65 | 5.20 | 11.20 | 13.20 | 7.55 | 6.30 | 10.30 | 12.35 | 8.18 | 22.79 | 14.92 | 5.06 | 9.92 | 18.44 | 4.95 | 6.64 | 179.5 | 10.30 | 10.30 | 7.80 | 9.29 | 4.73 | 14.64 | 10.57 |
|  | 27 | 37.80 | 16.15 | 47.95 | 33.65 | 37.80 | 43.90 | 107.2 | 56.50 | 69.90 | 47.85 | 22.31 | 37.92 | 19.50 | 34.22 | 34.03 | 35.62 | 12.98 | 25.96 | 68.70 | 121.3 | 53.40 | 15.61 | 45.72 | 29.72 | 23.92 | 39.04 |
|  | 29 | 27.00 | 11.31 | 12.25 | 13.10 | 39.80 | 38.30 | 20.05 | 29.20 | 20.60 | 22.40 | 21.02 | 23.12 | 25.37 | 27.64 | 35.72 | 32.18 | 7.91 | 12.05 | 49.50 | 14.10 | 16.10 | 2.87 | 18.12 | 12.25 | 16.62 | 13.63 |
|  | 30 | 44.80 | 87.45 | 34.60 | 54.60 | 35.40 | 57.70 | 43.20 | 55.80 | 49.00 | 25.40 | 30.02 | 26.12 | 22.96 | 13.78 | 26.27 | 12.28 | 13.74 | 16.17 | 40.70 | 22.00 | 13.00 | 24.31 | 12.30 | 5.53 | 20.32 | 12.86 |
|  | 32 | NA | NA | 26.00 | 44.70 | 61.20 | 18.40 | 66.50 | 79.15 | 40.50 | 42.70 | 21.80 | 23.15 | 24.62 | 28.68 | 26.61 | 22.28 | 18.84 | 17.00 | 46.40 | 25.20 | 25.80 | 57.94 | 20.52 | 12.44 | 25.02 | 13.99 |
|  | 33 | 20.90 | 25.45 | 8.25 | 7.65 | 12.10 | 9.20 | 14.85 | 16.85 | 6.85 | 6.90 | 12.49 | 26.66 | 13.20 | 8.02 | 9.92 | 14.61 | 12.39 | 10.00 | 8.02 | 6.20 | 6.80 | 2.97 | 4.98 | 7.96 | 9.02 | 11.19 |
|  | 34 | 20.85 | 23.00 | 75.60 | 36.25 | 34.80 | 65.05 | 121.7 | 68.30 | 87.60 | 13.20 | 27.78 | 28.50 | 36.00 | 34.57 | 45.16 | 35.77 | 36.66 | 36.13 | 66.60 | 46.50 | 46.70 | 33.50 | 53.91 | 23.30 | 43.27 | 37.41 |
|  | 35 | 25.10 | 30.50 | 25.20 | 27.60 | 45.00 | 14.90 | 82.70 | 87.70 | 64.85 | 22.65 | 24.68 | 29.27 | 46.69 | 33.18 | 38.45 | 22.32 | 26.72 | 21.58 | 26.60 | 24.60 | 25.40 | 2.29 | 43.72 | 24.52 | 37.21 | 40.62 |
|  | 36A | 53.85 | 16.90 | 48.45 | 19.90 | 18.10 | 15.75 | 43.20 | 39.55 | 72.45 | 12.95 | 16.78 | 11.62 | 8.94 | 13.78 | 19.93 | 24.52 | 27.06 | 12.47 | 18.20 | 33.90 | 18.20 | 14.79 | 9.93 | 16.91 | 24.87 | 28.53 |
|  | 37 | 51.30 | 31.65 | 30.30 | 33.80 | 62.80 | 16.85 | 28.90 | 31.10 | 92.70 | 11.30 | 45.82 | 40.00 | 22.90 | 52.94 | 69.14 | 34.48 | 29.26 | 9.09 | 14.10 | 15.40 | 14.80 | 22.49 | 21.46 | 8.58 | 56.34 | 61.47 |
|  | 38 | 28.70 | 27.45 | 29.70 | 25.95 | 66.20 | 11.80 | 35.40 | 50.55 | 77.50 | 22.25 | 27.11 | 35.60 | 26.30 | 38.73 | 38.04 | 16.52 | 31.61 | 36.86 | NA | NA | NA | 11.03 | 29.86 | 22.41 | 15.77 | 43.37 |
|  | 39 | 69.80 | 57.25 | 51.70 | 29.70 | 25.70 | 36.05 | 81.20 | 142.2 | 67.70 | 17.40 | 59.90 | 47.00 | 31.90 | 50.60 | 50.89 | 64.25 | 64.64 | 69.65 | 69.80 | 37.10 | 37.50 | 51.90 | 38.37 | 44.26 | 49.38 | 56.51 |
|  | 40 | 50.60 | 39.25 | 72.30 | 137.2 | 78.60 | 15.95 | 64.40 | 82.50 | 95.10 | 39.95 | 47.10 | 22.70 | 41.00 | 48.78 | 71.50 | 42.42 | 66.34 | 80.66 | 24.50 | 40.20 | 46.30 | 48.77 | 30.99 | 15.51 | 23.43 | 29.36 |
|  | 41 | 6.90 | 7.25 | 17.35 | 12.90 | 11.80 | 14.00 | 14.90 | 14.30 | 20.10 | 11.30 | 23.51 | 8.68 | 7.23 | 19.27 | 14.34 | 11.92 | 4.97 | 5.23 | 36.20 | 11.10 | 11.20 | 17.75 | 13.81 | 21.60 | 4.24 | 5.29 |
|  | 42 | 52.70 | 25.20 | 25.95 | 56.55 | 49.40 | 55.65 | 66.80 | 75.45 | 71.10 | 24.10 | 22.61 | 32.00 | 22.70 | 37.00 | 45.50 | 29.12 | 21.83 | 23.23 | 27.50 | 47.10 | 47.60 | 13.27 | 24.69 | 49.65 | 31.89 | 25.37 |
|  | 43 | 36.80 | 19.75 | 45.60 | 23.80 | 59.40 | 13.40 | 55.60 | 36.55 | 42.80 | 14.80 | NA | NA | NA | 15.98 | 23.27 | 20.14 | 27.86 | 7.07 | 27.50 | 18.30 | 18.50 | 17.79 | 11.13 | 14.48 | 20.18 | 15.05 |
|  | 45 | 66.75 | 33.30 | 12.05 | 37.65 | 31.60 | 12.20 | 25.80 | 64.10 | 34.20 | 90.60 | 42.18 | 88.64 | 17.76 | 29.23 | 46.17 | 28.20 | 26.72 | 15.98 | 88.60 | 37.10 | 37.40 | 24.31 | 32.28 | 21.02 | 38.47 | 51.01 |
|  | 46 | 51.55 | 24.80 | 51.40 | 34.80 | 20.55 | 46.20 | 48.70 | 76.40 | 83.30 | 45.70 | 55.67 | 43.57 | 43.68 | 30.76 | 29.00 | 27.94 | 34.30 | 22.15 | 29.00 | 115.5 | 115.5 | 2.54 | 37.13 | 26.28 | 112.4 | 85.37 |
|  | 48 | 74.50 | 34.35 | 43.40 | 38.40 | 40.20 | 10.50 | 20.00 | 29.45 | 68.45 | 15.20 | 23.48 | 35.88 | 26.42 | 32.35 | 69.14 | 19.24 | 59.93 | 7.77 | 24.50 | 13.10 | 24.50 | 64.39 | 65.55 | 22.15 | 48.84 | 41.32 |
| **TSS** | 1 | 10.0 | 200.0 | 15.0 | 44.0 | 18.0 | 225.0 | 74.0 | 27.0 | 10.0 | 18.0 | 8.0 | 5.0 | 14.0 | 12.0 | 10.0 | 10.0 | 388.0 | 84.0 | 28.0 | 28.0 | 30.0 | 86.0 | 62.0 | 34.0 | 3.0 | 6.0 |
|  | 2 | 10.0 | 32.6 | 10.0 | 67.0 | 39.0 | 323.0 | 38.0 | 18.0 | 10.0 | 19.0 | 7.0 | 15.0 | 15.0 | 16.0 | 12.0 | 15.0 | 270.0 | NA | 36.0 | 24.0 | 11.0 | 68.0 | 51.0 | 13.0 | 3.0 | 6.0 |
|  | 3 | 15.0 | 144.0 | 20.0 | 87.0 | 212.0 | 201.0 | 46.0 | 25.0 | 21.0 | 25.0 | 12.0 | 10.0 | 5.0 | 4.0 | 5.0 | 15.0 | 270.0 | 51.0 | 52.0 | 54.0 | 14.0 | 45.0 | 88.0 | 40.0 | 4.0 | 12.0 |
|  | 3A | 44.5 | 99.0 | 15.0 | 70.0 | 148.0 | 336.0 | 50.0 | 15.0 | 19.0 | 22.0 | 29.0 | 15.0 | 5.0 | 41.0 | 5.0 | 18.0 | 280.0 | 65.0 | 42.0 | 28.0 | 11.0 | 35.0 | 75.0 | 37.0 | 7.0 | 25.0 |
|  | 4 | 17.0 | 44.0 | 10.0 | 55.0 | 18.0 | 106.0 | 110.0 | 17.0 | 125.0 | 25.0 | 30.0 | 10.0 | 22.0 | 12.0 | 6.0 | 18.0 | 113.0 | 58.0 | 42.0 | 40.0 | 20.0 | 73.0 | 44.0 | 78.0 | 3.0 | 45.0 |
|  | 5 | 18.0 | 49.0 | 56.0 | 39.0 | 151.0 | 115.0 | 125.0 | 33.0 | 2,924 | 45.0 | 22.0 | 11.0 | 60.0 | 8.0 | 22.0 | 26.0 | 221.0 | 54.0 | 32.0 | 48.0 | 18.0 | 52.0 | 157.0 | 50.0 | 15.0 | NA |
|  | 6 | 27.0 | 38.0 | 15.0 | 25.0 | 25.0 | 52.0 | 112.0 | 52.0 | 11.0 | 90.0 | 20.0 | 20.0 | 52.0 | 14.0 | 16.0 | 30.0 | 131.0 | 79.0 | 64.0 | 22.0 | 25.0 | 28.0 | 43.0 | 35.0 | 17.0 | 20.0 |
|  | 7 | 43.0 | 201.0 | 22.0 | 37.0 | 66.0 | 27.0 | 23.0 | 105.0 | 65.0 | 25.0 | 17.0 | 17.0 | 5.0 | 32.0 | 10.0 | 48.0 | 24.0 | 30.0 | 112.0 | 98.0 | 116.0 | 26.0 | 40.0 | 43.0 | 18.0 | 11.0 |
|  | 8 | 27.0 | 45.0 | 16.0 | 24.0 | 25.0 | 15.0 | 20.0 | 23.0 | 19.0 | 21.0 | 24.0 | 15.0 | 16.0 | 4.0 | 9.0 | 42.0 | 19.0 | 9.0 | 78.0 | 12.0 | 13.0 | 7.0 | 13.0 | 13.0 | 9.0 | 11.0 |
|  | 8A | 267.0 | 57.0 | 15.0 | 30.0 | 172.0 | 92.0 | 58.0 | 75.0 | 28.0 | 76.0 | 106.0 | 71.0 | 98.0 | 20.0 | 43.0 | 72.0 | 157.0 | 24.0 | 135.0 | 146.0 | 152.0 | 23.0 | 62.0 | 36.0 | 18.0 | 43.0 |
|  | 9 | 41.0 | 62.6 | 22.0 | 32.0 | 31.0 | 57.0 | 35.0 | 108.0 | 17.0 | 48.0 | 40.0 | 36.0 | 39.0 | 28.0 | 51.0 | 68.0 | 37.0 | 146.0 | 122.0 | 18.0 | 43.0 | 14.0 | 172.0 | 15.0 | 32.0 | 28.0 |
|  | 10 | 18.0 | 20.0 | 15.0 | 25.0 | 15.0 | 12.0 | 30.0 | 39.0 | 18.0 | 40.0 | 24.0 | 15.0 | 5.0 | 8.0 | 11.0 | 32.0 | 19.0 | 17.0 | 45.0 | 40.0 | 47.0 | 27.0 | 52.0 | 13.0 | 8.0 | 7.0 |
|  | 12 | 105.0 | 25.0 | 28.0 | 990.0 | 56.0 | 58.0 | 353.0 | 447.0 | 284.0 | 56.0 | 199.0 | 69.0 | 950.0 | 63.0 | NA | 128.0 | 64.0 | 3,580 | 156.0 | 36.0 | 224.0 | 137.0 | 78.0 | 55.0 | 45.0 | 118.0 |
|  | 13 | 51.0 | 38.0 | 31.0 | 45.0 | 25.0 | 55.0 | 73.0 | 106.0 | 28.0 | 25.0 | 17.0 | 45.0 | 25.0 | 50.0 | 48.0 | 40.0 | 23.0 | 29.0 | 180.0 | 34.0 | 46.0 | 29.0 | 56.0 | 61.0 | 15.0 | 36.0 |
|  | 14 | 10.0 | 129.0 | 30.0 | 70.0 | 262.0 | 86.0 | 149.0 | 99.0 | 79.0 | 88.0 | 21.0 | 30.0 | 15.0 | 130.0 | 16.0 | 19.0 | 63.0 | 82.0 | 112.0 | 50.0 | 50.0 | 48.0 | 31.0 | 17.0 | 10.0 | 22.0 |
|  | 15 | 61.0 | 20.0 | 15.0 | 18.0 | 491.0 | 1,296 | 198.0 | 36.0 | 672.0 | 200.0 | 52.0 | 60.0 | 604.0 | 15.0 | 58.0 | 72.0 | 22.0 | 81.0 | 136.0 | 32.0 | 33.0 | 40.0 | 189.0 | 16.0 | 408.0 | 127.0 |
|  | 17 | 18.0 | 97.0 | 435.0 | 175.0 | 18.0 | 115.0 | 290.0 | 52.0 | 353.0 | 125.0 | 28.0 | 20.0 | 40.0 | 34.0 | 19.0 | 24.0 | 132.0 | 950.0 | 108.0 | 106.0 | 20.0 | 61.0 | 946.0 | 132.0 | 57.0 | 69.0 |
|  | 19 | 17.0 | 67.0 | 40.0 | 25.0 | 305.0 | 112.0 | 96.0 | 45.0 | 84.0 | 85.0 | 45.0 | 25.0 | 38.0 | 17.0 | 10.0 | 46.0 | 16.0 | 63.0 | 58.0 | 96.0 | 28.0 | 42.0 | 90.0 | 23.0 | 21.0 | 37.0 |
|  | 20A | 48.0 | 30.0 | 22.0 | NA | 48.0 | 18.0 | 46.0 | 55.0 | 25.0 | 25.0 | 18.0 | 35.0 | 20.0 | NA | 48.0 | 39.0 | 59.0 | 16.0 | 36.0 | 48.0 | NA | 51.0 | 48.0 | 45.0 | 42.0 | 56.0 |
|  | 21 | 25.0 | 20.0 | 20.0 | 55.0 | 31.0 | 97.0 | 65.0 | 39.0 | 25.0 | 70.0 | 36.0 | 20.0 | 15.0 | 7.0 | 63.0 | 19.0 | 10.0 | 55.0 | 64.0 | 32.0 | 12.0 | 43.0 | 148.0 | 24.0 | 37.0 | 70.0 |
|  | 22 | 25.0 | 35.0 | 25.0 | NA | 19.0 | 40.0 | 73.0 | 33.0 | 30.0 | 56.0 | 25.0 | 18.0 | 25.0 | 21.0 | 45.0 | 35.0 | 16.0 | 73.0 | 54.0 | 32.0 | 34.0 | 19.0 | 96.0 | 46.0 | 22.0 | 23.0 |
|  | 23 | 18.0 | 108.0 | 46.0 | 392.0 | 106.0 | 315.0 | 242.0 | 37.0 | 64.0 | 353.0 | 13.0 | 12.0 | 15.0 | 150.0 | 15.0 | 14.0 | 28.0 | 366.0 | 130.0 | 46.0 | 33.0 | 25.0 | 57.0 | 23.0 | 8.0 | 28.0 |
|  | 24 | 40.0 | 53.0 | 35.0 | 37.0 | 55.0 | 68.0 | 28.0 | 50.0 | 15.0 | 15.0 | 66.0 | 35.0 | 36.0 | 13.0 | 32.0 | 47.0 | 15.0 | 20.0 | 115.0 | 16.0 | 17.0 | 12.0 | 23.0 | 39.0 | 31.0 | 63.0 |
|  | 24A | 20.0 | 35.0 | 25.0 | 20.0 | 211.0 | 79.0 | 27.0 | 103.0 | 15.0 | 31.0 | 41.0 | 51.0 | 66.0 | 72.0 | 85.0 | 74.0 | 19.0 | 51.0 | 142.0 | 38.0 | 40.0 | 48.0 | 10.0 | 452.0 | 56.0 | 69.0 |
|  | 25 | 10.0 | 47.0 | 20.0 | 25.0 | 51.0 | 66.0 | 75.0 | 30.0 | 23.0 | 50.0 | 25.0 | 15.0 | 10.0 | 46.0 | 18.0 | 12.0 | 21.0 | 41.0 | 55.0 | 16.0 | 11.0 | 32.0 | 6.0 | 14.0 | 20.0 | 17.0 |
|  | 25A | 25.0 | 1,144 | 25.0 | 38.0 | 47.0 | 60.0 | 51.0 | 19.0 | 20.0 | 69.0 | 12.0 | 20.0 | 12.0 | 18.0 | 21.0 | 30.0 | 12.0 | 27.0 | 218.0 | 26.0 | 21.0 | 11.0 | 3.0 | 15.0 | 12.0 | 8.0 |
|  | 27 | 48.0 | 28.0 | 10.0 | 17.0 | 39.0 | 25.0 | 60.0 | 77.0 | 20.0 | 21.0 | 15.0 | 79.0 | 42.0 | 53.0 | 59.0 | 42.0 | 13.0 | 28.0 | 146.0 | 76.0 | 79.0 | 51.0 | 8.0 | 26.0 | 44.0 | 13.0 |
|  | 29 | 20.0 | 25.0 | 11.0 | 40.0 | 15.0 | 20.0 | 20.0 | 27.0 | 15.0 | 48.0 | 24.0 | 22.0 | 15.0 | 41.0 | 49.0 | 37.0 | 39.0 | 7.0 | 132.0 | 34.0 | 28.0 | 100.0 | 498.0 | 181.0 | 5.0 | 84.0 |
|  | 30 | 35.0 | 46.0 | 21.0 | 20.0 | 70.0 | 35.0 | 16.0 | 34.0 | 13.0 | 55.0 | 29.0 | 18.0 | 14.0 | 25.0 | 42.0 | 20.0 | 8.0 | 24.0 | 66.0 | 60.0 | 18.0 | 244.0 | 19.0 | 29.0 | 15.0 | 36.0 |
|  | 32 | NA | NA | 10.0 | 23.0 | 16.0 | 10.0 | 19.0 | 5.0 | 10.0 | 5.0 | 10.0 | 18.0 | 19.0 | 31.0 | 55.0 | 31.0 | 11.0 | 22.0 | 81.0 | NA | 45.0 | 61.0 | 10.0 | 48.0 | 56.0 | 9.0 |
|  | 33 | 15.0 | 157.0 | 20.0 | 28.0 | 25.0 | 25.0 | 25.0 | 10.0 | 24.0 | 83.0 | 23.0 | 36.0 | 11.0 | 25.0 | 14.0 | 31.0 | 114.0 | 81.0 | 25.0 | 17.0 | 14.0 | 79.0 | 47.0 | 15.0 | 1.0 | 12.0 |
|  | 34 | 36.0 | 22.0 | 59.0 | 53.0 | 25.0 | 56.0 | 82.0 | 90.0 | 17.0 | 12.0 | 43.0 | 58.0 | 78.0 | 55.0 | 138.0 | 47.0 | 13.0 | 60.0 | 124.0 | 40.0 | 73.0 | 50.0 | 19.0 | 54.0 | 56.0 | 34.0 |
|  | 35 | 73.0 | 45.0 | 20.0 | 30.0 | 40.0 | 30.0 | 89.0 | 100.0 | 15.0 | 28.0 | 35.0 | 28.0 | 35.0 | 79.0 | 42.0 | 28.0 | 6.0 | 77.0 | 88.0 | 6.0 | 14.0 | 62.0 | 41.0 | 45.0 | 32.0 | 81.0 |
|  | 36A | 159.0 | 42.0 | 790.0 | 18.0 | 33.0 | 95.0 | 209.0 | 35.0 | 8.0 | 65.0 | 245.0 | 427.0 | 638.0 | 632.0 | 59.0 | 38.0 | 8,380 | 18.0 | 120.0 | 142.0 | 47.0 | 19.0 | 102.0 | 101.0 | 157.0 | 21.0 |
|  | 37 | 40.0 | 35.0 | 27.0 | 20.0 | 130.0 | 76.0 | 25.0 | 39.0 | 17.0 | 65.0 | 58.0 | 85.0 | 19.0 | 76.0 | 89.0 | 64.0 | 158.0 | 61.0 | 136.0 | 6.0 | 42.0 | 149.0 | 39.0 | 50.0 | 61.0 | 19.0 |
|  | 38 | 20.0 | 115.0 | 28.0 | 15.0 | 25.0 | 47.0 | 32.0 | 63.0 | 28.0 | 50.0 | 55.0 | 25.0 | 19.0 | 69.0 | 75.0 | 46.0 | 22.0 | 73.0 | NA | NA | NA | 40.0 | 25.0 | 86.0 | 30.0 | 38.0 |
|  | 39 | 45.0 | 66.0 | 15.0 | 40.0 | 37.0 | 15.0 | 57.0 | 66.0 | 9.0 | 6.0 | 76.0 | 35.0 | 51.0 | 50.0 | 63.0 | 128.0 | 25.0 | 72.0 | 152.0 | 32.0 | 83.0 | 83.0 | 55.0 | 52.0 | 48.0 | 57.0 |
|  | 40 | 76.0 | 35.0 | 388.0 | 160.0 | 83.0 | 48.0 | 76.0 | 209.0 | 34.0 | 73.0 | 98.0 | 131.0 | 100.0 | 164.0 | 172.0 | 234.0 | 129.0 | 92.0 | 222.0 | 104.0 | 83.0 | 103.0 | 21.0 | 41.0 | 410.0 | 480.0 |
|  | 41 | 28.0 | 39.0 | 120.0 | 50.0 | 18.0 | 32.0 | 49.0 | 33.0 | 34.0 | 28.0 | 32.0 | 20.0 | 24.0 | 56.0 | 48.0 | 47.0 | 17.0 | 31.0 | 130.0 | 29.0 | 37.0 | 52.0 | 22.0 | 45.0 | 61.0 | 18.0 |
|  | 42 | 25.0 | 43.0 | 40.0 | 35.0 | 35.0 | 30.0 | 104.0 | 63.0 | 26.0 | 45.0 | 43.0 | 60.0 | 18.0 | 90.0 | 58.0 | 26.0 | 18.0 | 38.0 | 112.0 | 90.0 | 96.0 | 48.0 | 10.0 | 114.0 | 39.0 | 82.0 |
|  | 43 | 25.0 | 25.0 | 47.0 | 42.0 | 15.0 | 15.0 | 39.0 | 28.0 | 18.0 | 19.0 | NA | NA | NA | 23.0 | 37.0 | 25.0 | 9.0 | 15.0 | 112.0 | 10.0 | 25.0 | 14.0 | 2.0 | 14.0 | 17.0 | 23.0 |
|  | 45 | 71.0 | 36.0 | 32.0 | 35.0 | 64.0 | 40.0 | 140.0 | 91.0 | 23.0 | 62.0 | 93.0 | 45.0 | 37.0 | 39.0 | 74.0 | 120.0 | 29.0 | 21.0 | 144.0 | 42.0 | 63.0 | 53.0 | 25.0 | 51.0 | 43.0 | 74.0 |
|  | 46 | 20.0 | 20.0 | 23.0 | 45.0 | 25.0 | 16.0 | 27.0 | 17.0 | 17.0 | 45.0 | 18.0 | 15.0 | 25.0 | 34.0 | 24.0 | 33.0 | 10.0 | 17.0 | 164.0 | 121.0 | 125.0 | 96.0 | 5.0 | 47.0 | 20.0 | 39.0 |
|  | 48 | 102.0 | 38.0 | 170.0 | 25.0 | 80.0 | 55.0 | 21.0 | 31.0 | 15.0 | 10.0 | 67.0 | 25.0 | 30.0 | 61.0 | 82.0 | 50.0 | 49.0 | 51.0 | 40.0 | 22.0 | 132.0 | 56.0 | 45.0 | 48.0 | 29.0 | 54.0 |
